# Effectiveness and experiences of early intensive behavioral and naturalistic developmental behavior interventions for autism spectrum disorders: a mixed-methods systematic review and meta-analysis

**DOI:** 10.1186/s13034-025-00997-z

**Published:** 2025-12-26

**Authors:** Dong-Gyun Han, Yoonjae Lee, Hee-Sun Kim, Hyo-Weon Suh, Jeongeun Lee, Suk-Ho Shin, Moonbong Yang, Haemi Choi, Tae-Hyeong Kim, Jae-Gu Kang, Eunseol Ko, Jiyeon Lee, Min-Hyeon Park

**Affiliations:** 1 Department of Psychiatry, College of Medicine, Eunpyeong St. Mary’s Hospital, The Catholic University of Korea, Seoul, Republic of Korea; 2 Health Policy Research Team, Division of Healthcare Research, National Evidence-Based Healthcare Collaborating Agency, Seoul, Republic of Korea; 3 Dr. Shin’s Neuropsychiatric Clinic, Seoul, Republic of Korea; 4 Department of Special Education, Baekseok University, Seoul, Republic of Korea; 5 Department of Psychology, Ewha Womans University, Seoul, Republic of Korea

**Keywords:** Autism spectrum disorder, Applied behavior analysis, Naturalistic developmental behavior interventions, Early intensive behavior interventions, Treatment as usual, Systemic barriers

## Abstract

**Background:**

Autism spectrum disorder (ASD) is a neurodevelopmental condition marked by social communication deficits and restricted, repetitive behaviors. Among evidence-based practices (EBPs), interventions grounded in applied behavior analysis (ABA) principles—including Early Intensive Behavioral Intervention and naturalistic developmental behavioral interventions—are widely used. While the evidence suggests potential benefits, the findings are inconsistent, most studies carry a high risk of bias, and the quality of evidence is generally low to very low. Gaps also remain in comparisons with treatment as usual (TAU) and across intervention intensities.

**Aims:**

This mixed-methods systematic review and meta-analysis evaluated the quantitative effectiveness and qualitative experiences of ABA-based interventions for children and adolescents with ASD, addressing the methodological limitations of earlier studies, and examining comparisons with TAU.

**Methods:**

Seven databases were searched up to August 2023 following the PRISMA guidelines. Twenty-five studies met the inclusion criteria (16 randomized controlled trials, 9 qualitative). The quantitative outcomes included adaptive behavior, cognitive ability (IQ/DQ), language, daily living skills, socialization, joint attention, and autism symptom severity. Qualitative studies explored parents’ and practitioners’ experiences. Random-effects models were used, with subgroup analyses by intervention intensity and TAU comparisons.

**Results:**

The meta-analysis revealed significant improvements in adaptive behavior (SMD = 0.31, 95% CI: 0.04–0.59, GRADE = low), daily living skills (SMD = 0.36, 95% CI: 0.08–0.64, GRADE = low), language skills (SMD = 0.42, 95% CI: 0.24–0.60, GRADE = moderate), and joint attention behavior (SMD = 0.27, 95% CI: 0.04–0.49, GRADE = low) compared with the controls. High-intensity interventions had a notably greater effect on language skills (SMD = 0.72, 95% CI: 0.42–1.01) than low-intensity interventions (SMD = 0.34, 95% CI: 0.13–0.55). Comparisons with TAU revealed significant effects on adaptive behavior (SMD = 0.34, 95% CI: 0.02–0.66), daily living skills (SMD = 0.39, 95% CI: 0.07–0.71), and language skills (SMD = 0.51, 95% CI: 0.24–0.78). Qualitative findings highlighted perceived family and practitioner benefits but also barriers such as financial constraints and variability in training quality.

**Conclusion:**

This study confirms the effectiveness of ABA in improving developmental and behavioral outcomes in children with ASD. However, systemic challenges and variability in outcomes underscore the need for targeted policy initiatives, enhanced training programs, and further research on the impact of ABA on core ASD symptoms.

**Supplementary Information:**

The online version contains supplementary material available at 10.1186/s13034-025-00997-z.

## Introduction

 Autism spectrum disorder (ASD) is a neurodevelopmental disorder characterized by impairments in social communication and the presence of restricted, repetitive, and stereotyped behavioral patterns [1, 2]. With an estimated prevalence of 1 in 44 children [3], ASD represents a major public health concern because of its early onset, chronic trajectory, and lifelong impact. While the exact pathogenesis remains unclear, ASD is believed to result from the interaction between genetic and environmental factors [4–7]. Various interventions have been developed to address these challenges; however, their evaluation is inherently complex because of the heterogeneity of the population, developmental variability in symptom presentation, and persistent concerns regarding measurement validity and cost [8–10]. To address such complexity, mixed-methods approaches that integrate both quantitative and qualitative evidence have been recommended [11], but remain underutilized in prior reviews. Therefore, this study aimed to synthesize quantitative and qualitative findings from previous research on interventions for individuals with ASD.

In recent years, evidence-based practices (EBPs), defined as the integration of best available evidence with clinical expertise and client and family values and preferences [12], have gained increasing attention as validated approaches for supporting individuals with ASD [13]. Among these practices, interventions grounded in applied behavior analysis (ABA) principles—most notably structured interventions such as Early Intensive Behavioral Intervention (EIBI) and Discrete Trial Training (DTT), as well as Naturalistic Developmental Behavioral Interventions (NDBIs)—have been recognized as influential models that systematically apply behavioral strategies to address socially significant behaviors and early developmental goals [14, 15]. These approaches have been widely adopted in both research and practice; however, their effectiveness has not been uniformly consistent across studies.

Conceptually, EBPs are often classified into two categories: focused intervention practices, which target specific skills or behaviors, and comprehensive program models, which integrate multiple practices to address broader developmental domains [13, 16–19]. Although these two approaches differ in scope, they are grounded in the same behavioral principles and are often applied in combination in clinical and educational settings [13, 20]. In this review, we explicitly employ the term *“ABA-based interventions”* to encompass both focused intervention practices and comprehensive program models that systematically apply the principles of ABA, which is consistent with the terminology proposed by Schreibman et al. [15]. This definition includes NDBIs such as the Early Start Denver Model (ESDM) and Pivotal Response Treatment (PRT). While specialists in these approaches often regard them as distinct from ABA, Vivanti et al. [21] emphasize that they are nonetheless rooted in core ABA principles. Therefore, for the purposes of the present study, we use the term “*ABA-based interventions*” consistently, but note that terminological differences exist across the field.

Several studies have reported that ABA-based interventions can lead to improvements in various domains, including cognitive ability, adaptive behavior, receptive and expressive language, communication, daily living skills, and socialization [22–26]. Moreover, systematic reviews and meta-analyses have reported substantial variability in effect sizes, raising questions about the overall magnitude and reliability of these effects [22, 24, 25, 27, 28]. Importantly, a recent Cochrane review by Reichow et al. [29] concluded that although EIBI may yield small improvements, the certainty of evidence was low and the risk of bias high, with concerns including randomization bias and broader methodological limitations, underscoring the need for cautious interpretation and further high-quality syntheses.

Beyond these concerns, challenges at the structural and implementation levels persist. Despite the emergence of telehealth-delivered and parent-mediated formats designed to improve accessibility [30–32], ABA-based interventions remain constrained by financial demands, workforce shortages, and variability in training quality [33, 34]. Such barriers may undermine treatment fidelity and contribute to divergent outcomes, independent of the theoretical soundness of the intervention. Moreover, relatively few studies have directly compared ABA-based interventions with standard treatments such as treatment as usual (TAU). Although some recent study have incorporated subgroup comparisons with TAU [22], these efforts are narrow in scope and often restricted to structured models such as EIBI, while developmentally informed approaches such as NDBIs that target early developmental goals are excluded. Consequently, the inconsistent findings across the literature may reflect not only variability in the intrinsic effects of ABA-based interventions and their implementation but also the absence of systematic evidence contrasting ABA-based interventions with TAU.

Taken together, these considerations underscore the need for integrative research that captures a broad spectrum of outcomes and directly compares ABA-based interventions with TAU. To this end, the present study adopted a mixed-methods approach that synthesizes quantitative evidence on the effectiveness of ABA-based interventions with qualitative research exploring the experiences of parents, therapists, and institutional infrastructures. A meta-analysis was conducted to examine intervention outcomes across dimensions such as intervention intensity and subgroup-specific contrasts, with a particular focus on comparisons with TAU. By integrating quantitative and qualitative findings, this study aimed to provide balanced and comprehensive evidence to inform clinical practice and policy decisions regarding timely and individualized early interventions for individuals with ASD.

## Methods

### Search strategy

We followed the Preferred Reporting Items for Systematic Reviews and Meta Analyses (PRISMA) 2020 guidelines [35]. The procedures of this study were disclosed in the PROSPERO international prospective register of systematic reviews (No. CRD42023466097) before data extraction began to minimize the possibility of reporting bias [36, 37]. The inclusion criteria for the population, intervention, comparison, outcome, time, and study design (PICO-TS) of the present study were as follows: (1) population: children and young adults with ASD (aged 0–24) for quantitative review and caregivers of parents with ASD, hospital-based institutes, and intervention practitioners for qualitative review. The broader age range (0–24 years) was chosen to reflect the application of ABA-based interventions beyond early childhood and to ensure consistency between the quantitative and qualitative evidence included in this review; (2) intervention: behavioral interventions grounded in the core principles of ABA and designed to support early developmental goals in individuals with ASD. This includes both traditional structured approaches and developmentally informed, naturalistic models (e.g., NDBI frameworks). The selection was guided by interventions that are empirically supported, age-appropriate, and conceptually coherent as early intervention strategies; (3) comparison: language therapy, conventional therapy, occupational and play therapy, psychoeducation intervention, wait-list, and TAU. For the purpose of this review, TAU was defined a priori to encompass standard community or clinical services routinely provided to children with ASD. Eligible studies were included if their TAU conditions comprised one or more of the following: occupational and physical therapy, speech–language interventions, the Treatment and Education of Autistic and related Communication-handicapped Children (TEACCH) program, the Developmental, Individual Difference, Relationship-based (DIR) model, play therapy, or home-based infant–toddler sessions. TAU could also involve parent guidance, family therapy, social-skills training, cognitive behavioral therapy, pharmacotherapy as indicated, as well as developmental preschool programs and related services supplemented by community referrals and resource manuals; (4) outcomes: adaptive behavior, autism symptom severity, and cognitive ability (e.g., intelligence quotient [IQ], development quotient [DQ]), socialization, daily living skills, receptive and expressive language, joint attention behavior, and gross motor skills for quantitative review, as well as the experiences and perspectives of caregivers and practitioners regarding ABA-based interventions for qualitative review; (5) time (follow-up period): no restrictions; (6) study design: the quantitative studies included randomized controlled trials (RCTs), whereas the qualitative studies included narrative studies, case studies, grounded theory, phenomenology, and ethnography, as well as qualitative components of mixed-methods studies.

The following types of studies were excluded: (1) studies whose design was not RCTs; (2) studies that did not focus specifically on ABA-based interventions aligned with early developmental goals or that applied ABA techniques only as part of broader, eclectic programs in which the effects of ABA-based strategies could not be meaningfully isolated or evaluated; (3) studies that did not use standardized or semistructured outcome measurements; (4) studies in which efforts to obtain mean and standard deviation values were unsuccessful because author correspondence yielded no response and the study lacked the necessary descriptive or statistical details for calculation; and (5) studies published in languages other than English or Korean or those that did not appear in peer-reviewed journals or doctoral dissertations as well as gray literature, abstracts, and unpublished materials. Seven databases—PubMed, EMBASE, the Cochrane Library, PsycINFO, CINAHL, KoreaMed, and Kmbase—were systematically searched with a combination of the key terms “ABA”, “applied behavior analysis”, “NDBI”, “Naturalistic Developmental Behavior Interventions”, “EIBI”, “Early Intensive Behavior Intervention”, “autism”, and “autism spectrum disorder” to obtain relevant publications. The detailed search terms can be found in the supplementary sections, eTable 1.

### Study selection

All identified records retrieved through the search strategy were imported into *Covidence* (systematic review software) [38, 39], where duplicates were automatically removed. Two reviewers (DGH and YL) independently screened the remaining records in two stages: (1) title and abstract review to exclude studies that clearly did not meet the inclusion criteria and (2) full-text assessment of potentially relevant articles to determine final eligibility. Any discrepancies between the two reviewers were resolved through discussion and when necessary, adjudicated by a third reviewer (JL) to ensure transparency and consistency. The entire study selection process was documented in accordance with the PRISMA guidelines and summarized in the PRISMA flow diagram [35].

### Quality assessment

The quality of each study and the certainty of evidence were assessed by two reviewers (DGH and YL) via the Cochrane Risk of Bias tool (ROB) [40]. Six domains were assessed as components of the ROB: the randomization process, derivation for intended interventions, missing outcome data, measurement of the outcome, selection of the reported results, and overall results. The degree of bias was classified as low, moderate, or high. To evaluate the consistency of reviewer judgments, inter-rater reliability was calculated using Cohen’s Kappa, which quantifies the level of agreement beyond chance; the κ value was 0.83, indicating strong agreement between reviewers [41]. Discrepancies were resolved through discussion until consensus was achieved. Graphical illustrations were generated to visually summarize the distribution of risk of bias across domains.

Furthermore, the certainty of the body of evidence for each outcome was assessed using the Grading of Recommendations Assessment, Development, and Evaluation (GRADE) approach [42]. This framework evaluates evidence quality across five domains—risk of bias, inconsistency, indirectness, imprecision, and publication bias—and classifies the overall certainty as high, moderate, low, or very low. Importantly, the use of GRADE enhances the transparency and reproducibility of systematic reviews and provides a structured basis for developing clinical guidelines and policy recommendations, thereby linking research evidence directly to decision-making in practice and health policy [43].

Qualitative articles were also assessed by the same authors via the Critical Appraisal Skills Program (CASP) Qualitative Checklist [44]. This tool is widely recognized for its reliability in evaluating qualitative research. The appraisal was based on the following ten criteria: a clear statement of the research aims (Q1), appropriateness of the qualitative methodology (Q2), suitability of the research design to the aims (Q3), appropriateness of the recruitment strategy (Q4), adequacy of data collection methods in addressing the research issue (Q5), consideration of the researcher–participant relationship (Q6), attention to ethical issues (Q7), rigor of data analysis (Q8), clarity of the findings (Q9), and overall value of the research (Q10). The inclusion of studies was determined through ongoing discussion and reflection during the appraisal process. To enhance the reliability of qualitative appraisals, inter-rater agreement was assessed. The analysis yielded a Cohen’s Kappa of 0.81, indicating strong concordance between the two reviewers [41]. Any discrepancies were resolved through discussion until consensus was achieved.

### Data extraction

For the quantitative review, data extraction was initially performed by one author (DGH) and subsequently cross-checked for accuracy by two additional authors (YL and JL). A standardized set of data extraction items, as recommended by PRISMA 2020 [35], was employed to ensure consistency across the included studies. From each eligible RCT, the following information was systematically collected: study characteristics (author, year, journal, and country); participants’ characteristics (including age and biological sex); details of the experimental intervention (type of program, session length, frequency per week, and overall duration); the nature of the control condition; provider characteristics (e.g., therapist, teacher, or parent); and the context in which the intervention was delivered (e.g., individual or group setting, home, preschool, or clinic). In addition, study funding sources and conflicts of interest (COI) were documented, together with all reported outcome domains and corresponding measurement instruments, as well as information on intervention intensity (in hours per week) and duration (in weeks). Intervention intensity was defined as a dichotomous variable, which was proposed by previous research [24, 45], whereby interventions delivering 20 or more hours per week were classified as high intensity, and those delivering less than 20 h per week were classified as low intensity. Any discrepancies in data extraction were resolved through discussion among the authors.

As part of the qualitative review process, two reviewers (DGH and YL) held a preparatory meeting to pilot the data collection procedure before data extraction. To resolve discrepancies, all reviewers (DGH, YL, and JL) met at the end of each stage, whereas clarification of missing or unclear data was sought by contacting the authors at most twice. Data extraction was independently conducted by two reviewers in two phases on the basis of the standardized methodology recommended by the Joanna Briggs Institute (JBI) [46]. In the first phase, information on study characteristics (author, country, year, journal, methodology for data collection and analysis), study context and setting, demographic details of participants (age, biological sex), phenomena of interest, authors’ conclusions, and aspects related to research rigor was collected. In the second phase, the study findings and their supporting illustrations were extracted and assigned a reliability level categorized as “unequivocal,” “equivocal,” or “unsupported” [46]. These procedures were designed to ensure transparency and methodological rigor throughout the process [47].

### Data synthesis and analysis

For the meta-analyses, forest plots were used to assess variability visually on the basis of confidence intervals. Heterogeneity was assessed using standard statistics [48]. The *Q* test evaluates whether between-study variance exceeds chance, although it can be oversensitive with large samples. The *I*² statistic quantifies the proportion of variance due to heterogeneity (0–25% negligible, 25–50% low, 50–75% moderate, >75% high). *τ*² estimates the absolute between‐study variance, and *H*² expresses the relative excess variance, with values near 1 indicating little heterogeneity. Together, these measures provide a comprehensive evaluation of heterogeneity in meta-analyses [48]. When the heterogeneity was moderate to high (*I*² ≥ 50%) and a sufficient sample size (*n* >10) was included, sensitivity analyses were conducted to explore potential sources of heterogeneity [49]. These analyses included assessing changes in effect estimates by excluding studies with a high influence on effect size or heterogeneity identified through Baujat plots and systematically excluding each included study to evaluate the impact on the effect estimates [49]. The possibility of publication bias was assessed when feasible (*n* >10) by combining graphical methods such as funnel plots with statistical tests such as Egger’s test [50, 51].

Considering the heterogeneity across studies, a random-effects model using a restricted maximum-likelihood estimator was applied. Effect estimates are reported as standardized mean differences (SMD) with 95% confidence intervals, and the significance level was set at 5% [52]. This study included several cases in which a single outcome was measured via multiple assessments. Treating these as separate outcomes posed the risk of overestimating variance. To mitigate this risk, the mean effect size and combined standard error (SE) were calculated via formulas proposed by Borenstein and colleagues [53]. Building on prior research suggesting that intervention intensity may substantially influence effect sizes [22], we conducted subgroup analyses based on intensity levels in addition to comparisons with TAU. All analyses were performed via Stata/SE 18.1 (Stata Corp LLC, College Station, TX) and R (version 3.6.3, http://cran.r-project.org/).

For the qualitative meta-synthesis, we employed meta-aggregation method recommended by JBI [54, 55]. Meta-aggregation is used to integrate qualitative evidence to complement quantitative syntheses, such as meta-analyses of RCTs, particularly when findings appear inconsistent [56]. Unlike meta-ethnography, realist synthesis, or thematic synthesis, meta-aggregation emphasizes fidelity to original findings, thereby reducing interpretive distortion and yielding synthesized statements that can inform evidence-based policy and guideline development [54]. This approach, therefore, provides a useful means of clarifying the discrepancies observed in prior research on ABA-based interventions for individuals with ASD. Given that the study relied exclusively on secondary analysis of published literature and did not involve human participants, ethical approval was not needed.

As described in the protocol [54, 55], all qualitative findings were systematically extracted along with their supporting illustrations. Only findings classified as unequivocal or equivocal were retained for synthesis, whereas unsupported statements were excluded. The extracted findings were then organized into categories, with each category requiring at least two supporting findings to ensure consistency. These categories were subsequently integrated into synthesized findings through an inductive process, based on similarities in meaning. The entire synthesis was conducted by consensus among the three reviewers (DGH, YL, and JL), involving repeated readings and iterative discussions to achieve agreement and strengthen the reliability of the results.

**Fig. 1:**
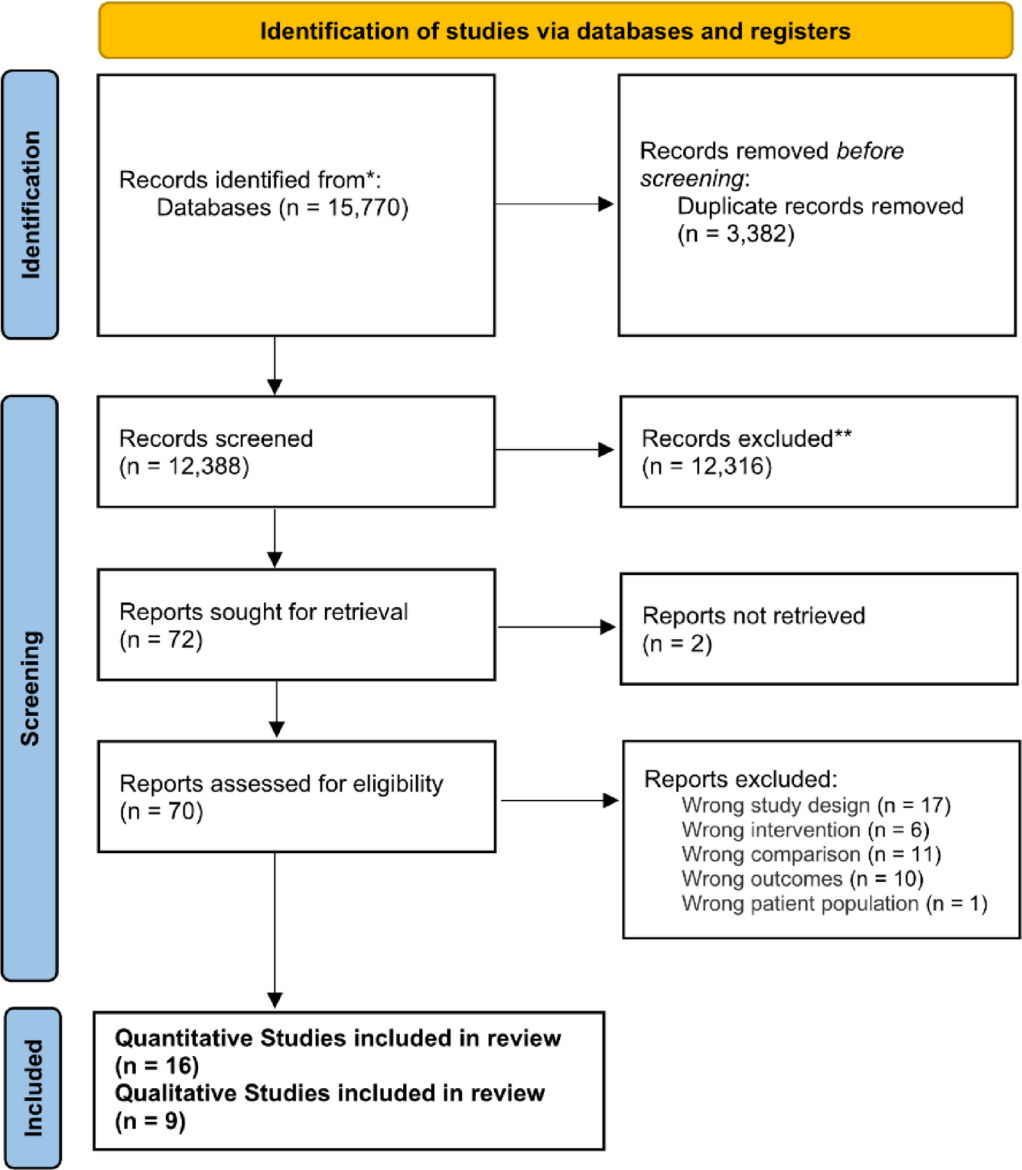
PRISMA flow chart

## Results

### Eligible studies and their characteristics

A PRISMA flowchart summarizing the article selection process is presented in Fig. 1. The initial database search identified 15,770 studies. After the removal of duplicates, 12,388 studies remained and were screened for inclusion. Following a review of the titles and abstracts, 70 studies were selected, and after a full-text review, 25 studies were included in the final analysis. The list of selected studies along with the list of excluded studies and the reasons for their exclusion are provided in eTable 1.

Across 16 quantitative studies, a total of 893 individuals with ASD were included, with reported ages ranging from 12 months to 15 years. The specific age ranges for each included study are detailed in Table 1. All participants were diagnosed with ASD by clinicians via the Autism Diagnostic Observation Schedule (ADOS), the Autism Diagnostic Interview-Revised (ADI-R), or the Diagnostic and Statistical Manual of Mental Disorders Fourth Edition (DSM-IV) or Fifth Edition (DSM-5) [57–60] (Table 1).

**Table 1:** Characteristics of included quantitative studies

Study	Study design	Participants characteristics	Experiment group	Comparison condition	Intervention	Outcomes	Intensity	Duration	Settings
Chang et al. (2016)	RCT	*n* = 66 (89% males), Age range: 3–5 years	JASPER	Waitlist (continued with the usual preschool curriculum only and received JASPER 4 months later)	• Daily program with in vivo coaching sessions (A maximum of 60, 15 min long coaching sessions) • Two initial 30-min preparation sessions without the children were conducted in addition to the 8-week in vivo coaching sessions • Providers: Professionals	RL (MSEL), EL (MSEL), JA (ESCS, TCX)	Low	8 weeks	Individual and group
Dawson et al. (2010)	RCT	*n* = 48 (77.77% males), Age range: 18–30 months	ESDM	TAU	• 20 h/week (1 session of 2 h, twice a day, 5days/week) with parents training • Parent delivery for 5 or more hours/week of ESDM • Whatever community services the parents chose were added • Providers: Trained therapists and parents	AB (VABS), SC (VABS), DL (VABS), RL (MSEL), EL (MSEL), MS (VABS), ASS (ADOS)	High	2 years	Individual
Dekorte et al. (2021)	RCT	*n* = 44 (70.45% males), Age range: 9–15 years	PRT	TAU (ranging from 1.5 h/week to 1 h/month)	• 12 weekly sessions, each 45 min PRT session, except for one teacher session which included a 90 min school visit (if applicable) (7 parent–child sessions, 3 parent-only sessions, and 2 sessions in which the child’s teacher was involved) • Extended with 8 weeks, when participants were rated as not “much improved” or “very much improved” on the Clinical Global Impression- Improvement scale (4 parent–child sessions, 2 parent-only sessions, and 1 session with the teacher were added) • Providers: Clinician	AB (VABS-II), SC (VABS-II), DL (VABS-II), ASS (ADOS-II)	Low	12 weeks or 20 weeks	Individual
Dixon et al. (2021)	RCT	*n* = 28 (85.71% males), Age range: 3–13 years	ABA	Waitlist	• Traditional-ABA intervention Experimental Group (T-ABA group): Number of programs selected for each participant to work on at a time varied across individuals. • Comprehensive-ABA intervention Experimental Group (C-ABA group) • Providers: One to two therapists	IQ (WPPSI-IV, WISC-V)	Low	12 weeks	Individual (Clinic)
Gengoux et al. (2019)	RCT	*n* = 43 (88.4% males), Age range: 2–5 years)	PRT	Waitlist and stable community treatments	• Week 1 to 12: ‘intensive phase’ (Parent training session of 60 min/week, children received home treatment 10 h/week from clinician) • Week 12 to 24: ‘maintenance phase’ (Parent training session of 60 min/month, children received home treatment 5 h/week) • Providers: Clinician and parents	SC (SRS-II), EL (VABS-II, PLS-5, MSEL, MCDI 396, MCDI 680)	Low	24 weeks	Individual (Home)
Goods et al. (2013)	RCT	*n* = 15, Age range: 3–5 years	JASPER	TAU (30 h/week)	• Two 30-minute sessions/week for 12 weeks • Providers: Graduate students in educational psychology	EL (RDLS), RL (RDLS), JA (ESCS)	Low	12 weeks	Individual (Preschool)
Hardan et al. (2015)	RCT	*n* = 48 (75% males), Mean age: 4.1 years	PRT	Psychoeducation (1 session/week, 10 sessions were 90 m parent-only group meetings, and 2 were 60 m individual meetings)	• 1 session/week (8 weeks of 90 min parent-only group sessions, 4 weeks of 60 min parent-child dyads sessions) • Providers: Parents-only	SC (SRS), EL (VABS-II, PLS-4, MCDI 396, MCDI 680), RL (VABS)	Low	12 weeks	Group
Kasari et al. (2014)	RCT	*n* = 112 (83% males), Age range: 2–5 years	JASPER	Caregiver educated module (not TAU)	• 21 h sessions/week • Providers: Parents-only (caregivers)	JA (ESCS)	Low	12 weeks	Individual (Home)
Kasari et al. (2015)	RCT	*n* = 86 (81% males), Mean age: 31.5 (22–36 months)	JASPER	Parents-only psychoeducational intervention	• Two sessions of 30 min/week or 1 h/week • Providers: Parents-only	JA (PCX), EL (RDLS), RL (RDLS)	Low	10 weeks	Individual
Lawton et al. (2012)	RCT	*n* = 16, Age range: 3–5 years	JASPER	Waitlist	• Children enrolled half-day • Providers: Teachers	JA (ESCS)	Low	6 weeks	Individual (Preschool)
Mohammadzaheri et al. (2022)	RCT	*n* = 20 (100% males), Age range: 6-12years	PRT	TAU (standard language intervention)	• 3 sessions/week, 1 session of 1 h • Providers: Speech and language pathologist	EL (MLU)	Low	2 months	Individual (center)
Panganiban et al. (2022)	RCT	*n* = 54 (95% males), Age range: 3–5 years	JASPER	TAU (Standard curriculum as usual)	• Embedding JASPER strategies into curriculum, and classrooms ran four and a half hours daily, 5days/week • Providers: Teachers	EL (MSEL), RL (MSEL), JA (ESCS)	Low	None reported	Group (Preschool)
Rogers et al. (2012)	RCT	*n* = 98 (77.5% males), Age range: 12–24 months	ESDM	TAU	• 1 session of 1 h/week with parents sessions • Providers: Parents-only	AB (VABS-II), SC (VABS-II), DL (VABS-II), EL (MCDI Voca production), RL (MCDI Voca comprehension, MCDI parases understood), DQ (MSEL)	Low	12 weeks	Individual (Clinic)
Rogers et al. (2019)	RCT	*n* = 118 (78% males), Age range: 14–24 months	ESDM	TAU	• Phase 1: − 1 session of 1 h/week - Providers: Parents • Phase 2: - Children: 20 h/week - Primary caregiver(s): 2 h of parent coaching every 2 weeks - Providers: Trained staff members	AB (VABS-II), ASS (ADOS), DQ (MSEL)	High	Phase 1: 12 weeks, Phase 2: 24 months	Phase 1: Individual (Clinic) Phase 2: Individual (home and occasionally daycare or preschool)
Sullivan et al. (2014)	RCT	*n* = 48 (77% males) Age range: 18–30 months	ESDM	TAU	• 30 h/week - 1 session of 2 h individually with child, twice a day, 5days/week - An additional parents deliver 10 h intervention/week • Providers: Parents and therapist	EL (MSEL), RL (MSEL)	High	2 years	Individual
Vernon et al. (2019)	RCT	*n* = 23 (87% males) Age range: 18–56 months	PRT	Waitlist	• 10 h/week (8 h of one-on-one clinician-implemented treatment, 2 h of parent education) • Sessions delivered in home and community settings • Providers: Clinician and parents	AB (VABS-II), SC (VABS-II), DL (VABS-II), EL (MSEL, PLS-5), RL (MSEL, PLS-5), ASS (ADOS-II), MS (VABS-II)	Low	6 months (26 weeks)	Individual and group (Clinic)

ABA = Applied Behavior Analysis; AB = Adaptive Behavior; ADOS = Autism Diagnostic Observation Schedule; ASS = Autism Symptom Severity; DL = Daily Living Skills; DQ = Developmental Quotient; EL = Expressive Language; ESCS = Early Social Communication Scales; ESDM = Early Start Denver Model; IQ = Intelligence Quotient; JA = Joint Attention; JASPER = Joint Attention, Symbolic Play, Engagement, and Regulation; MCDI = MacArthur-Bates Communicative Development Inventories; MCDI-PU = MacArthur-Bates Communicative Development Inventory: Phrases Understood; MCDI-VC = MacArthur-Bates Communicative Development Inventory: Vocabulary Comprehension; MCDI-VP = MacArthur-Bates Communicative Development Inventory: Vocabulary Production; MLU = Mean Length of Utterance; MSEL = Mullen Scales of Early Learning; MS = Motor Skills; PLS = Preschool Language Scale; PRT = Pivotal Response Training; RCT = Randomized Controlled Trial; RDLS = Reynell Developmental Language Scales; RL = Receptive Language; SC = Socialization; SRS = Social Responsiveness Scale; TAU = Treatment as Usual; TCX = Teacher-Child Interaction; VABS = Vineland Adaptive Behavior Scales; WISC = Wechsler Intelligence Scale for Children; WPPSI = Wechsler Preschool and Primary Scale of Intelligence

Among all the studies, three were categorized as high intensity [61–63], whereas the others were identified as low intensity [64–76]. Eight studies utilized TAU as the control condition, five adopted a waitlist design, and the remaining three employed other interventions, such as psychoeducation. The studies included 7 professional-only interventions, 5 that combined parents and professionals, and 4 that included only parents. The duration of the interventions varied widely and ranged from 6 weeks to 2 years.

Among the 25 studies, nine were qualitative or mixed-method studies that focused on ABA-based interventions [77–84]. The selected studies explored diverse populations, including children with ASD, their parents, and practitioners. Participants were recruited from various settings, such as home-based programs, disability day care facilities, and community services. The sample sizes ranged from two families to more than 100 participants, including multidisciplinary professionals shown in Table 2.

**Table 2:** Characteristics of included qualitative studies

Study	Study design	Participants and settings	Purpose	Focus of inquiry	Major findings	Implications
Anderson et al. (2017)	• Interview	• 2 mothers practicing intensive ABA home training for their child in late kindergarten age with ASD. • 2 families randomly selected among the families practicing intensive home training in Copenhagen.	• To investigate why parents decide to train their child with disabilities at home, considering the controversial idea of intensive home training.	• Explore parents’ motivation for ABA training, their child’s developmental progress (developmental time), and social activity involvement before and after initiating home training (social agency). • The interview guide followed a chronological sequence: (a) pre-training phase, (b) decision-making process, (c) home training phase, and (d) future considerations.	• Choosing intensive ABA home training was a response to the lack of developmental support in conventional kindergarten settings, where limited participation impacted children’s development and well-being. • The decision to begin training stemmed from broader concerns about the child’s overall developmental environment, not just individual rehabilitation. • The primary motivation was to promote the child’s development and create a supportive local environment. • Home training gave mothers a sense of control over their child’s well-being and developmental time. • Despite gaining a sense of control, mothers expressed concerns about potential challenges in the child’s future school environment.	• The study emphasizes the need for improved developmental support in conventional education settings to reduce reliance on intensive home training. • While home training empowered parents by giving them a sense of control, concerns about future schooling remain. • Future efforts should focus on integrating supportive measures into mainstream education and addressing parental needs for involvement.
Bang. (2021)	• Thematic analysis of qualitative data and semi-structured in-depth interview	• 6 staff providing direct services to adults with develop-mental disabilities at day care facilities in Seoul and Gyeonggi Province.	• To explore perceptions and application of ABA among practitioners providing services to adults in a disability day care facility.	• What types of disabilities and problem behaviors are displayed by adults in disability day care facilities? • How do practitioners in these facilities perceive the application and effectiveness of ABA? • What support needs and individual efforts are required to implement ABA in such settings?	• Intellectual disability and ASD were the most common disabilities in the facilities, with aggressive behaviors frequently observed. • Practitioners recognized ABA as an effective method for reducing aggression, increasing adaptive behaviors, and improving service users’ quality of life. • Positive outcomes led to a shift in staff perceptions, highlighting ABA’s importance. • However, successful implementation requires adequate staffing, financial support, and expertise. • Collaboration between institutions and government, along with addressing personal limitations and physical demands on staff, is essential for effective application.	• Financial support is essential for implementing ABA in disability day care facilities and should focus on staffing and facility needs. • Training ABA specialists requires significant time and cost, suggesting a solution where the government partially subsidizes retraining for social workers, who would then serve at facilities for a set period. • Additionally, financial support should address structural improvements or expansions to meet long-term facility needs.
Boyd et al. (2001)	• Mixed-methods (Case study with parents survey)	• 22 children primarily diagnosed with autism or PDD-NOS and participated in an EIBI program	• To assess the outcome of EIBI and systematically evaluate the data, specifically whether the favorable recovery rates and anticipated treatment reduction observed in controlled environments can be replicated within the community setting	• Assess whether a child could be considered ‘recovered’ from autism based on: 1) Intellectual disability status. 2) Dependence on specialized education and support after EIBI. 3) Parental views and satisfaction with the EIBI program. • Case Review Analyzed final progress reports, current IEPs, and recent psychological evaluations. • Parent Questionnaire Focused on parental satisfaction with the EIBI program and its outcomes.	• While community-based EIBI yielded meaningful outcomes, no participants achieved full recovery and continued to require specialized services. • The best responders to EIBI were placed on medication following the termination of the program. • Some findings raised doubts about the effectiveness of intensive treatment: no significant differences were observed in behavioral measures between the EIBI group and an ad hoc comparison group that received traditional services instead of EIBI. • Despite these concerns, parents expressed high satisfaction with the implementation of EIBI and the improvements observed in their children (though it remains unclear compared to what these outcomes were deemed satisfactory).	• Parental satisfaction with EIBI may result from its structured, intensive, home-based support, such as parental guidance and instructional control, which complement the treatment’s limited developmental outcomes. • Further research is needed to compare EIBI with other ABA interventions.
Dillenburger et al. (2004)	• Mixed-methods (Case study with parents survey)	• 4 parents of children diagnosed with ASD or Asperger syndrome receiving home-based ABA in Northern Ireland.	• To explore parents’ perceptions of ABA outcomes, including its impact on the child, family life, and parental confidence and empowerment.	• Effectiveness of ABA in achieving developmental and behavioral goals for children. • Parent education’s role in treatment intensity and outcome. • Impact of treatment age and duration on success.	• Parents reported high satisfaction with ABA outcomes, including improvements in children’s behaviors, family dynamics, and parental empowerment. • Parent education enhanced treatment intensity and efficacy. • Late treatment onset remained effective from a parental perspective. • Short-term parent education also led to significant results.	• Parent education in ABA is crucial for achieving better outcomes and may increase treatment intensity. • Future research should focus on direct assessments of parental implementation and factors influencing outcomes in older children and long-term interventions.
Dillenburger et al. (2012)	• Mixed-methods (Case study with parents survey)	• 95 parents of 100 children diagnosed with ASD. • 67 multidisciplinary professionals in Northern Ireland. The study examined current education services and future needs.	• To investigate the current state and improvement needs of educational services for children with ASD. • To analyze the necessity and experiences of ABA-based interventions.	• The need for and current status of ABA-based education and home interventions. • Perspectives on the effectiveness and feasibility of ABA from parents and professionals. • The role of parents and institutional support in implementing ABA.	• A severe lack of ABA-based education and certified staff forces parents to implement home-based ABA programs. • Parents are better informed about ABA than professionals and more actively seek new information. • Professionals often misunderstand ABA as a narrow treatment and acknowledge the need for further training. • Limited availability of ABA-based schools and high financial burdens pose challenges for families.	• A balanced, inclusive approach to treatment selection should emphasize parental participation and choice. • Governments should actively support certified ABA training and integration into public education. • ABA, as a behavioral science, plays a critical role in ASD education and treatment, requiring enhanced collaboration between parents and professionals.
Grindle et al. (2009)	• Semi-structured interview	• 53 parents of children diagnosed with ASD participated in the study.	• To explore parents’ experiences and perceptions of EIBI programs, including its impact on family life, emotional well-being, and practical challenges.	• The effects of EIBI on family dynamics, including parental emotional well-being and sibling experiences. • Practical challenges in implementing EIBI, including therapist management and administrative burdens.	• Parents reported positive effects of EIBI on their child, family, and personal empowerment but faced challenges like stress from managing therapists and privacy loss. • Over one-third of parents felt disappointed with limited progress due to unrealistic expectations. • Siblings benefited from involvement in EIBI but also required emotional support. • Parental stress was linked to program demands and marital strain, highlighting the need for emotional and practical support systems.	• Service providers should expand their focus to include family support alongside child-centered treatment. • Initiatives like therapist training, parent education, and emotional support (e.g., ACT-based interventions) could mitigate challenges and enhance family well-being. • Future research should explore why some families discontinue EIBI and its impact on both parents and children.
McPhilamy and Dillenburger. (2013)	• Mixed-methods (Thematic analysis of qualitative survey data)	• 15 families (17 children: 15 boys, 2 girls) with ASD participated. • 20 families were in Northern Ireland, and three in Italy. • 10 families were using ABA, while five had discontinued after sufficient progress.	• To examine parents’ experiences with ABA-based interventions, its impact on their child and family. • To explore systemic challenges and the need for public support.	• The effectiveness of ABA on child outcomes (e.g., communication, behavior, and independence) and family quality of life. • Barriers to accessing ABA-based interventions, including lack of public support and professional endorsement.	• ABA-based interventions significantly improved children’s communication, reduced challenging behaviors, and enhanced family quality of life. • Parents reported high satisfaction with ABA but criticized the lack of public funding and professional support. • Many professionals were less informed about ABA than parents, which hindered its broader adoption. • The expense of private ABA was a major barrier for families, despite its proven cost-effectiveness compared to lifelong care.	• Publicly funded education systems should adopt ABA-based interventions to ensure equitable access for families. • Re-education of professionals in ABA principles is necessary to make informed recommendations and policies. • Policies should emphasize individualized, evidence-based approaches to maximize each child’s potential.
Park. (2016)	• Narrative analysis and semi-structured in-depth interviews	• 4 mothers and their children (ages 6–26) with ASD or Asperger syndrome who received over 2 years of ABA in the United States.	• To examine the impact of ABA-based intervention on families with ASD children, focusing on changes in the quality of life for both the child and the family. • To identify challenges faced by families during the intervention’s implementation.	• Changes in the child’s life and mother’s rearing attitudes and behaviors. • Changes in interactions between the child and family members, and in the aspect of family stress. • Overview of implementation including challenges and experiences of interventionists.	• Participants reported positive changes, including improvements in the child, the family, and new relationship formation. • Six factors were identified as key to successful intervention despite adaptation challenges: 1) Active caregiver participation. 2) Intervention providers with cultural sensitivity who understand the child’s unique traits and parents’ perspectives. 3) Collaborative team efforts to improve skills, independence, and quality of life. 4) Systematic data recording and database management. 5) Consistent and ongoing intervention participation. 6) Public support and funding to reduce the financial burden on parents.	• The practicality of ABA interventions is closely tied to the qualities of interventionists. • Korean policymakers should consider introducing ABA-based interventions on a public scale, addressing the lack of systematic support despite existing evidence. • Therapists must understand the child’s traits and apply flexible, tailored interventions. • South Korea’s legal frameworks support intervention, but increasing demand for behavioral interventions for ASD calls for reevaluation of the educational system and budget allocation.
Tzanakaki et al. (2012)	Semi-structured interview	• 30 mothers of children with autism (31 children: 28 boys, 3 girls) in the UK, aged 49–82 months. • 16 families used University-supervised EIBI programs, and 14 used private service providers.	• To explore how parents decide to implement EIBI programs, their experiences during this process, and systemic challenges in accessing information and support.	• The decision-making process for implementing EIBI. • Accessibility of information and support from professionals. • Barriers to services and funding for EIBI programs.	• Parents often learned about EIBI from other parents, the internet, or books, rather than professionals. • Unrealistic expectations about outcomes (e.g., “normal functioning”) were common due to over-optimistic information sources. • Many families faced funding disputes and self-financed interventions. • Lack of collaboration among diagnostic and educational professionals created barriers for parents.	• Parents need honest, up-to-date information about EIBI outcomes and duration. • Professionals should provide better guidance on evidence-based interventions during the diagnostic process. • Collaboration between diagnostic, educational, and intervention services should be strengthened to improve accessibility and continuity of care for families.

ABA, Applied Behavior Analysis; ACT, Acceptance and Commitment Therapy; ASD, Autism Spectrum Disorder; EIBI, Early Intensive Behavioral Intervention; FAN-Q, Family Autism Needs Questionnaire; FQOL, Family Quality of Life; IEP, Individualized Education Plan; NI, Northern Ireland; PDD-NOS, Pervasive Developmental Disorder-Not Otherwise Specified; QUB, Queen’s University Belfast; SD, Standard Deviation

The included studies examined key aspects of ABA-based interventions, such as their effectiveness in improving developmental and behavioral outcomes, parents’ and practitioners’ experiences, and systemic barriers such as funding and professional training. The methodologies included interviews, thematic analyses, and case studies, with some studies incorporating parent surveys and practitioner feedback. These diverse study designs provide a comprehensive understanding of the multifaceted impact of ABA and highlight critical areas for further research and policy development. For all included studies, conflicts of interest and funding sources were assessed, and these details are presented in eTable 4.

### Measurement of outcomes

In this study, the selected outcomes for measuring ASD symptoms included adaptive behavior, daily living skills, socialization, receptive and expressive language skills, joint attention behavior, the severity of autism, motor skills, IQ, and DQ (Table 1).

Adaptive behavior was assessed using the Adaptive Behavior Composite (ABC) score of the Vineland Adaptive Behavior Scales (VABS I or II) [85, 86], which provides a standardized summary of overall adaptive functioning derived from three domains: daily living skills, socialization, and communication. Adaptive behavior was assessed in five studies, with four using the VABS-I and one using the VABS-II. In addition to the composite score, we also analyzed the domain subscores separately to capture specific functional outcomes. Daily living skills were further evaluated via the VABS, and socialization via both the VABS and the Social Responsiveness Scale (SRS I or II) [85–88]. Daily living skills were measured in four studies, including three with the VABS-I and one with the VABS-II. Socialization was evaluated in six studies, comprising three that used the VABS-I, one that used the VABS-II, and two that used the SRS-I.

Receptive and expressive language skills were measured via the Mullen Scales of Early Learning (MSEL), VABS, Preschool Language Scales, Fourth and Fifth Editions (PLS-4, PLS-5), MacArthur-Bates Communicative Development Inventories (CDI), and Reynell Developmental Language Scales (RDLS) [85, 89–93]. We also included the measurement of the mean length of utterance (MLU) as part of expressive language skills via a semistructured approach [94], as cited in [72]. For receptive language, composites were generated in Rogers et al. [75] and Vernon et al. [76], whereas single-instrument assessments included four studies using the MSEL, two using the RDLS, and one using the VABS-I. For expressive language, composite estimates were calculated when multiple instruments were applied within a single study [67, 70, 76], while single-instrument measures included four studies with the MSEL, two with the RDLS, one with the MLU, and one with the CDI Voca Production subscale.

Additionally, joint attention behavior was assessed via the Early Social Communication Scale (ESCS) and the Caregiver‒Child Interaction Coding System (including teachers and parents) [95–97]. Joint attention behavior was reported in six studies, with one combining Teacher‒Child Interaction (TCX) and ESCS, four using the ESCS alone, and one using the Parents‒Child Interaction (PCX). The severity of autism symptoms was evaluated with the ADOS and ADOS-2 [59, 98, 99], reported as calibrated severity scores (CSS; range 1–10). Autism symptom severity was assessed in four studies, two employing the ADOS-I and two employing the ADOS-II. Motor skills were measured via the VABS I and II [85, 86], whereas IQ and DQ were assessed via the Wechsler Intelligence Scale for Children (WISC-IV) and MSEL [89, 100]. Specifically, composite estimates were statistically derived for expressive language [67, 70, 76], receptive language [75, 76], and joint attention [64] using the mean effect size and combined SE formulas as described in the *Methods* section.

**Fig. 2:**
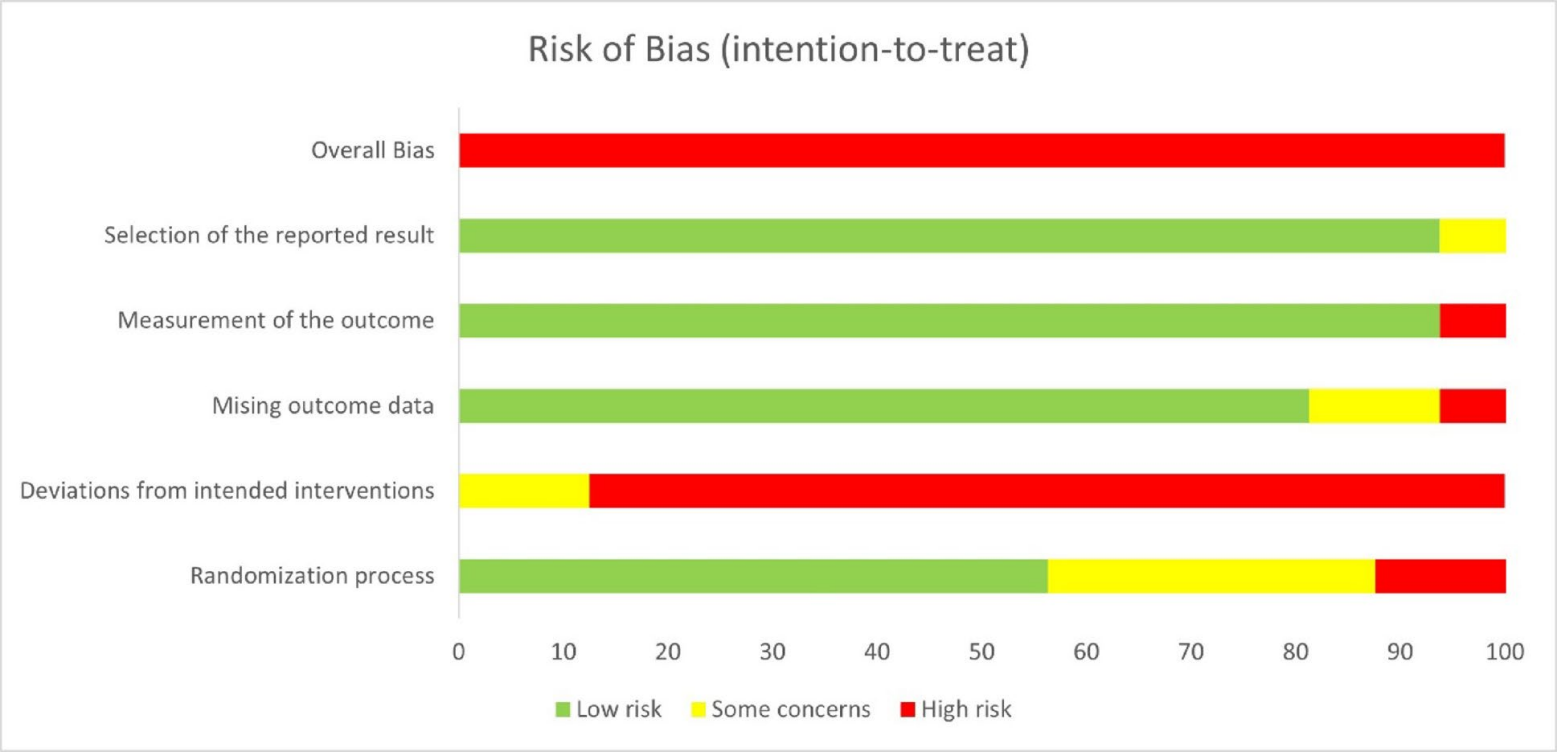
Risk of bias for all included outcomes

### Risk of bias appraisal

For the quantitative studies, the risk of bias was assessed independently for all included studies in Fig. 2 and eTable 3. In five studies, the randomization method or allocation concealment procedures were reported with insufficient clarity and were therefore classified as “some concerns” [61, 63, 72, 75, 76], whereas in two studies, concealment was entirely absent and thus rated as “high risk of bias” [64, 68]. With respect to deviation from the intended intervention criterion, two studies were marked as subject to some concerns [64, 68], and all remaining studies were considered to have a high risk of bias because of the lack of blinding of the participants and personnel to the intervention status [61, 65–67]. With respect to the missing outcome data criterion, two studies were found to have some concerns [61, 75], whereas only one study was deemed to have a high risk of bias [68]. Additionally, one study was rated as having a high risk of bias for the measurement of outcomes [76]. Finally, a single study was noted to be subject to some concerns regarding bias in the selection of the reported results [74]. Visual inspection of the funnel plots revealed no evidence of significant publication bias (eFigure 4). Egger’s test for funnel-plot asymmetry was not performed for individual outcomes, as each included fewer than ten studies in accordance with the Cochrane Handbook Sect. 13.3.5.4 [51].

For qualitative studies, the CASP Qualitative Checklist was utilized. Nine of these studies were rated as having a low risk of bias across all the items [77–84, 101], except for Item 7. Owing to ethical considerations in Item 7, two studies were classified as “cannot tell” [79, 84] (eFigure 2).

### Quality of evidence

#### Study limitations

Under the GRADE framework, the evidence was downgraded owing to pervasive methodological deficiencies. More than half of the randomized trials failed to adequately describe sequence generation and allocation concealment procedures [61, 63, 72, 75, 76], and two trials did not implement any concealment measures [64, 68]. Blinding of participants and personnel was largely absent, raising concerns about potential performance bias [61, 65–67]. Several studies reported differential or excessive attrition, with one trial exceeding acceptable levels and others demonstrating imbalance between groups [61, 68, 75]. In one trial, outcome assessment procedures lacked sufficient rigor [76], and in another, the reported analyses did not clearly align with the prespecified protocol [74]. Collectively, these shortcomings in trial design, conduct, and reporting warranted a downgrade for study limitations in our GRADE assessment.

#### Consistency

For the outcome of autism symptom severity, the *I*² statistic exceeded 50%, indicating substantial heterogeneity and rendering it difficult to ensure consistency in study quality. In contrast, all the other outcomes met the predefined heterogeneity thresholds, thereby confirming their consistency.

#### Directness

All included studies focused exclusively on children and adolescents with ASD; therefore, these findings are generalizable to the broader population of children and adolescents with ASD.

#### Precision

Except for the 16 studies assessing language skills (*n* = 549), all the other outcome domains had sample sizes less than 400 and thus failed to meet the precision criterion.

#### Publication bias

Although publication bias cannot be entirely ruled out, no serious bias was detected for any of the outcomes.

#### GRADE system

The quality of evidence according to the GRADE system is summarized in Table 3. As a consequence of important limitations in study design, inconsistency and lack of precision, the GRADE assessment classified the evidence for language skills as moderate quality, that for autism symptom severity as very low quality, and that for all other outcomes as low quality (Table 4).

**Table 3:** GRADE evidence profiles

Quality assessment^a^								Effect SMD (95% CI)	Quality	Importance
Outcome domain	No of studies	No of patients	Risk of bias	Inconsistency	Indirectness	Imprecision	Publication bias			
Adaptive behavior	5	331	Serious limitations	No serious inconsistency	No serious indirectness	Serious imprecision^c^	Undetected	SMD = 0.31, 95% CI: 0.04–0.59	⊕⊕○○	Critical
Socialization	6	304	Serious limitations	No serious inconsistency	No serious indirectness	Serious imprecision^c^	Undetected	SMD = 0.30, 95% CI: 0.02–0.58	⊕⊕○○	Critical
Daily living skills	4	213	Serious limitations	No serious inconsistency	No serious indirectness	Serious imprecision^c^	Undetected	SMD = 0.36, 95% CI: 0.08–0.64	⊕⊕○○	Critical
Language skills	16	549	Serious limitations	No serious inconsistency	No serious indirectness	No serious imprecision	Undetected	SMD = 0.49, 95% CI: 0.29–0.68	⊕⊕⊕○	Critical
Joint attention behavior	6	283	Serious limitations	No serious inconsistency	No serious indirectness	Serious imprecision^c^	Undetected	SMD = 0.27, 95% CI: 0.04–0.49	⊕⊕○○	Critical
Autism symptom severity	3	233	Serious limitations	Serious inconsistency^b^	No serious indirectness	Serious imprecision^c^	Undetected	SMD = 0.73, 95% CI: −0.06 to 1.51	⊕○○○	Critical

*SMD* standard mean difference, *CI* confidence interval

^a^The study design was not described in detail, given that all included studies were randomized controlled trials

^b^Quality was rated down for inconsistency because *I*^2^ > 50%

^c^Quality was rated down for imprecision due to total population size is less than 400

GRADE Working Group grades of evidence:

- High certainty (⊕⊕⊕⊕): We are very confident that the true effect lies close to that of the estimate of the effect

- Moderate certainty (⊕⊕⊕○)

: We are moderately confident in the effect estimate; The true effect is likely to be close to the estimate of the effect, but there is a possibility that it is substantially different

- Low certainty(⊕⊕○○): Our confidence in the effect estimate is limited: The true effect may be substantially different from the estimate of the effect

- Very low certainty(⊕○○○): We have very little confidence in the effect estimate: The true effect is likely to be substantially different from the estimate of effect

**Table 4:** Synthesis of findings based on qualitative data

Synthesis	Categories
1. Perceived Impact and Practical Utility of ABA	1.1 Observable behavioral progress in children
	1.2 Positive reappraisal of ABA’s effectiveness
	1.3 Reservations about effectiveness and expectations
2. Burden, Exhaustion, and Role Conflicts in Implementation	2.1 Overwhelming responsibilities and emotional fatigue
	2.2 Internal tension between caregiving and control
3. Institutional Barriers and Resource Constraints	3.1 Limited funding and poor governmental support
	3.2 Shortage of trained staff and program discontinuity
	3.3 Need for public support and equitable access
4. Ethical Identity and Advocacy among Stakeholders	4.1 Strong parental/staff advocacy and resilience
	4.2 Frustration from lack of authority and autonomy

### Results of the quantitative review

A meta-analysis of 16 RCTs revealed that ABA-based interventions had positive therapeutic effects on the domains of adaptive behavior, daily living skills, socialization, expressive and receptive language skills, joint attention behavior, and autism symptom severity. Unfortunately, the outcomes for IQ, DQ, and motor skills could not be included in the meta-analysis because the number of studies available for these outcomes was two or fewer. Additionally, an evaluation of the funnel plot revealed no significant asymmetry across all outcomes, except for language skill outcomes (for which a sensitivity analysis was conducted, excluding the relevant studies) (eFigure 3–4).

**Fig. 3:**
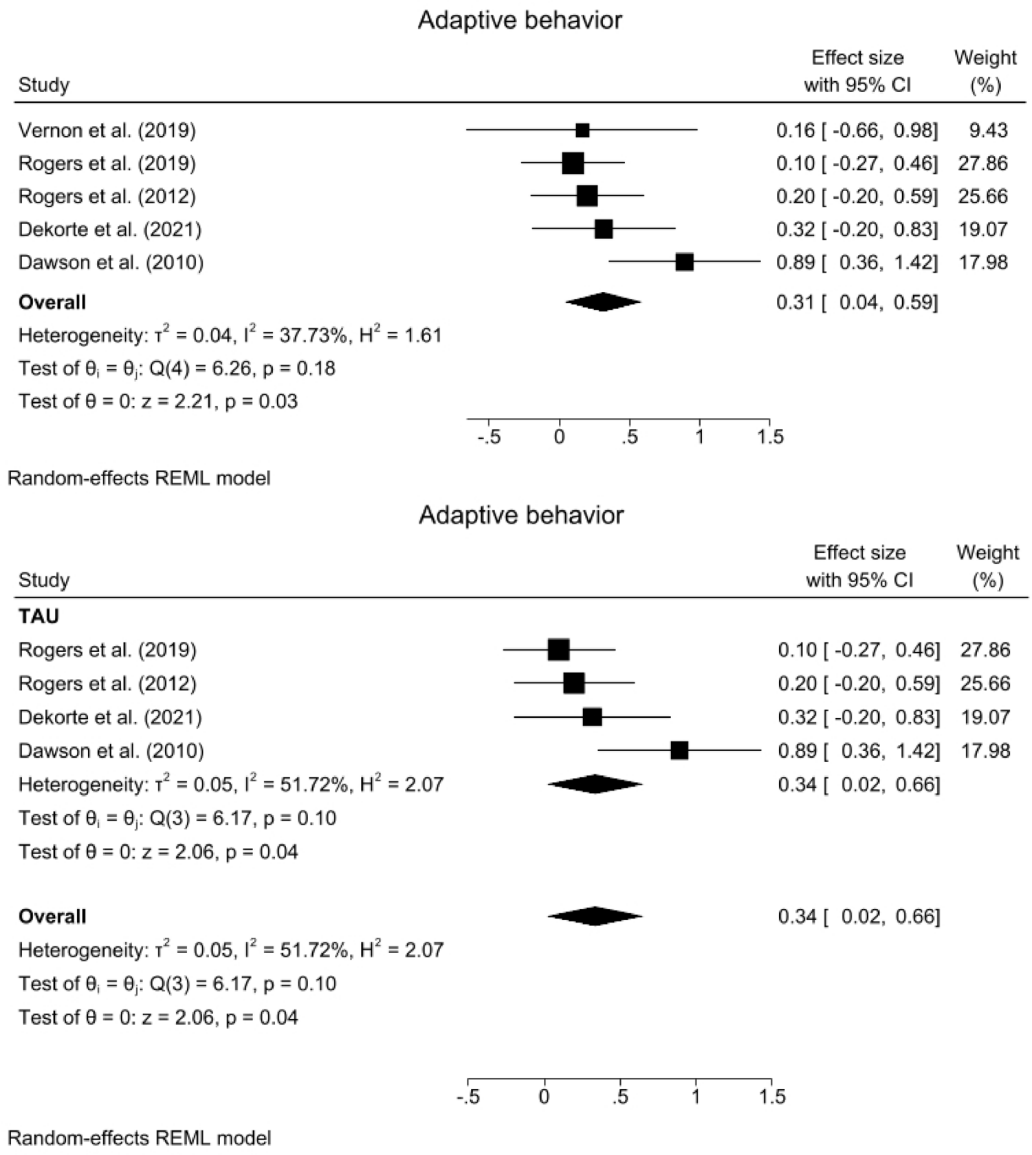
Forest plots for adaptive behavior. **a** Overall analysis of five studies. **b** Subgroup analysis of four studies comparing interventions with TAU. The non-TAU subgroup (one study) is not shown separately. *TAU* Treatment as Usual, *CI* confidence Interval

#### Adaptive behavior

A random effects meta-analysis revealed that compared with the control interventions, the ABA-based interventions significantly improved adaptive behavior (SMD = 0.31, 95% CI: 0.04 ~ 0.59, z = 2.21, *p* = 0.03). The heterogeneity was nonsignificant and small in magnitude (*Q* = 6.17, *p* = 0.10, *I*^2^ = 51.72%, GRADE = low) (Fig. 3a).

Compared with TAU, ABA interventions had a significant effect on adaptive behavior (SMD = 0.34, 95% CI: 0.02 ~ 0.66, z = 2.06, *p* = 0.04), with nonsignificant heterogeneity (*Q* = 6.26, *p* = 0.18, *I*^2^ = 37.73%) (Fig. 3b). A subgroup analysis of treatment intensity for adaptive behavior was not performed because none of the subgroups included the minimum required number of three studies.

**Fig. 4:**
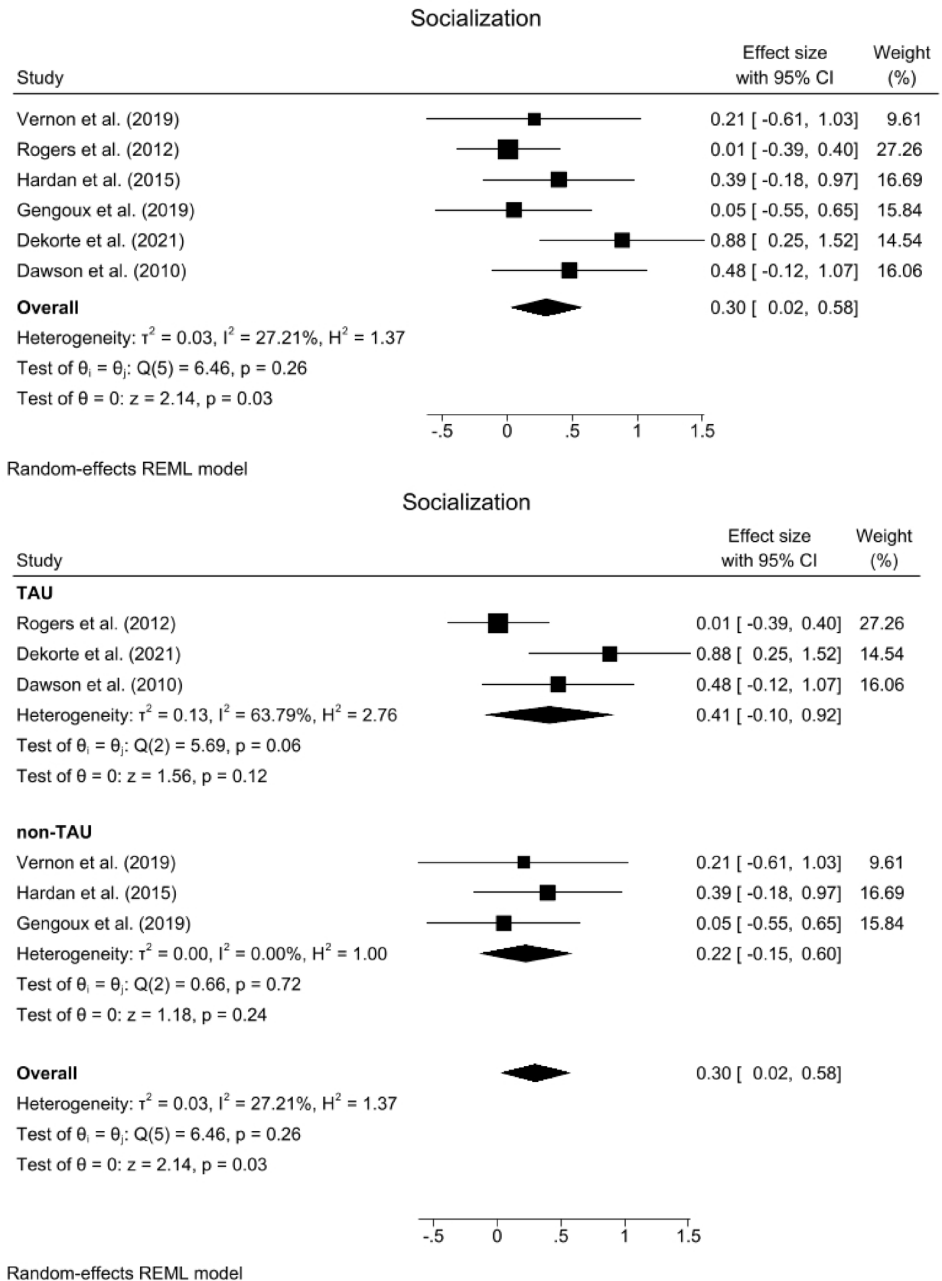
Forest plots for socialization. **a** Overall effect. **b** Subgroup analysis for comparison with the TAU group. *TAU* treatment as usual, *CI* confidence Interval

#### Socialization

The results from the random effects meta-analysis indicated that compared with the control interventions, the ABA-based interventions significantly improved socialization (SMD = 0.30, 95% CI: 0.02 ~ 0.58, z = 2.14, *p* = 0.03). The heterogeneity was nonsignificant and small in magnitude (*Q* = 6.46, *p* = 0.26, *I*^2^ = 27.21%, GRADE = low) (Fig. 4a).

Compared with TAU, ABA interventions had no effect on socialization (SMD = 0.41, 95% CI: −0.10 ~ 0.92, z = 1.56, *p* = 0.12), with nonsignificant heterogeneity (*Q* = 5.69, *p* = 0.06, *I*^2^ = 63.79%). Compared with those in the non-TAU group, the group differences were not significant (*Q* = 0.33, *p* = 0.57) (Fig. 4b). Subgroup analysis of the treatment intensity for socialization was not performed because none of the subgroups included the minimum required number of three studies.

**Fig. 5:**
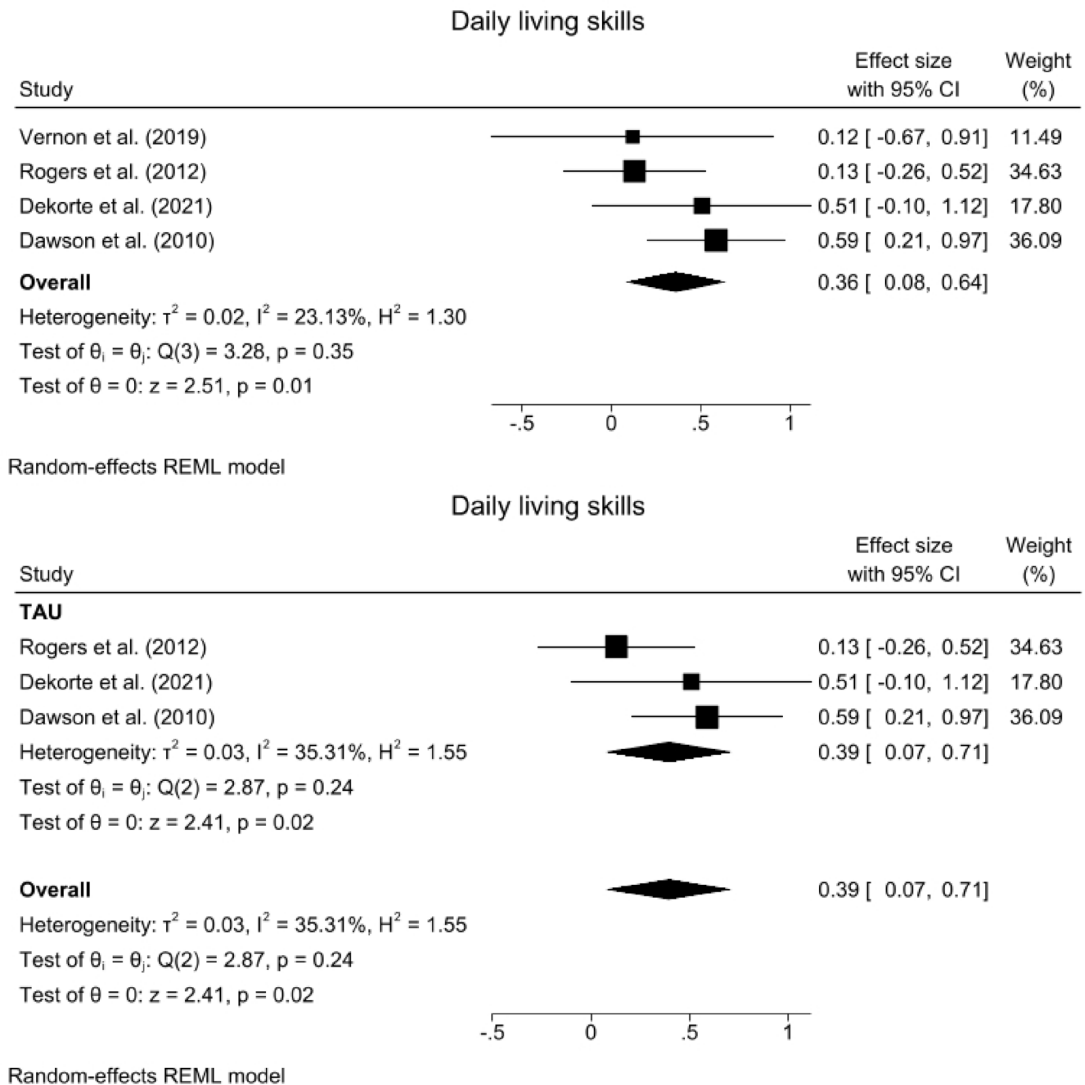
Forest plots for daily living skills. **a** Overall effect. **b** Subgroup analysis for comparison with the TAU group. *TAU* Treatment as usual, *CI* confidence Interval

#### Daily living skills

A random-effects meta-analysis revealed that compared with the control interventions, the ABA-based interventions had a significant effect on daily living skills (SMD = 0.36, 95% CI: 0.08 ~ 0.64, z = 2.51, *p* = 0.01). Heterogeneity was nonsignificant and small in magnitude (*Q* = 3.28, *p* = 0.35, *I*^2^ = 23.13%, GRADE = low) (Fig. 5a).

Compared with the TAU intervention, the ABA intervention had a significant effect on daily living skills (SMD = 0.39, 95% CI: 0.07 ~ 0.71, z = 2.87, *p* = 0.02), with nonsignificant heterogeneity (*Q* = 2.87, *p* = 0.24, *I*^2^ = 35.31%) (Fig. 5b). A subgroup analysis of treatment intensity for daily living skills was not performed because none of the subgroups included the minimum required number of three studies.

**Fig. 6:**
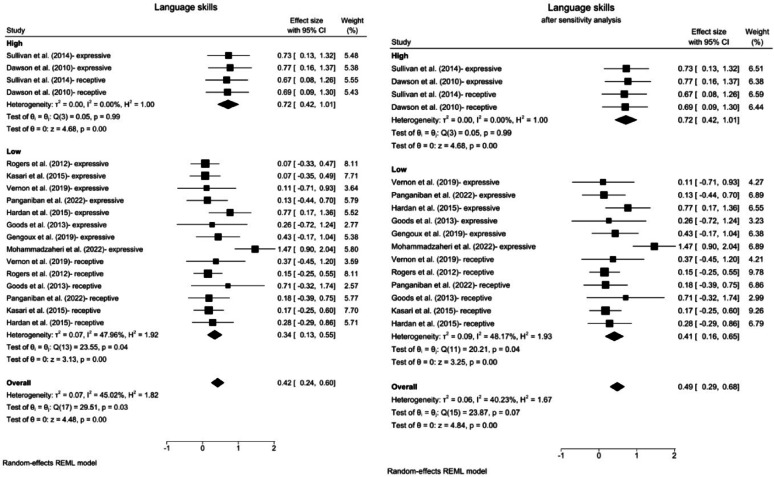
Forest plots for language skills. **a** Overall effect and subgroup analysis for the intensity level. **b** Results after excluding outliers via sensitivity analysis. *CI* confidence Interval

#### Receptive and expressive language skills

The results of a random effects meta-analysis revealed that compared with the control interventions, the ABA-based interventions significantly affected overall receptive and expressive language skills (SMD = 0.42, 95% CI: 0.24 ~ 0.60, z = 4.48, *p* < 0.001). The heterogeneity was significant and moderate in magnitude (*Q* = 29.51, *p* = 0.03, *I*^2^ = 45.02%, GRADE = moderate) (Fig. 6a).

With respect to treatment intensity, the subgroup analysis revealed a significant effect for both the high-intensity (SMD = 0.72, 95% CI: 0.42 ~ 1.01, z = 4.68, *p* < 0.001) and low-intensity treatment (SMD = 0.34, 95% CI: 0.13 ~ 0.55, z = 3.13, *p* < 0.001). However, heterogeneity was significant and moderate in magnitude for low intensity studies (*Q* = 23.55, *p* = 0.04, *I*^2^ = 47.96%), whereas high-intensity studies revealed nonsignificant heterogeneity (*Q* = 0.05, *p* = 0.99, *I*^2^ < 1%).

Given the significant overall heterogeneity and the inclusion of more than 10 studies, a sensitivity analysis was conducted to exclude studies identified as outliers [69, 75]. ABA-based interventions continued to demonstrate a significant therapeutic effect with an increased overall effect size (SMD = 0.49, 95% CI: 0.29 ~ 0.68, z = 4.84, *p* < 0.001). As all outlier studies were categorized as low intensity, the results for the high-intensity treatment remained unchanged, whereas the effect size for the low-intensity treatment increased significantly (SMD = 0.41, 95% CI: 0.16 ~ 0.65, z = 3.25, *p* < 0.001). Following the sensitivity analysis, overall heterogeneity was resolved and became statistically nonsignificant (*Q* = 23.87, *p* = 0.07, *I*²=40.23%). Additionally, the heterogeneity for the low-intensity treatment showed little to no change (*Q* = 20.21, *p* = 0.04, *I*²=48.17%) (Fig. 6b).

**Fig. 7:**
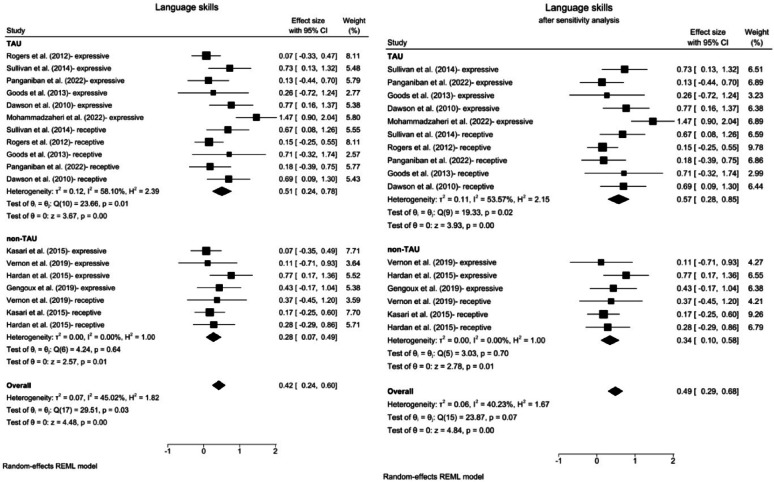
Forest plots for language skills. **a** Subgroup analysis for comparisons with the TAU group. **b** Results after excluding outliers by sensitivity analysis. *TAU* treatment as Usual, *CI* confidence Interval

Compared with the TAU intervention, the ABA intervention had a significant effect on language skills (SMD = 0.51; 95% CI: 0.24 ~ 0.78; z = 3.67; *p* < 0.001), with significantly moderate heterogeneity (*Q* = 23.66, *p* = 0.01, *I*^2^ = 58.10%) (Fig. 7a). Even after the identified outlier was excluded through sensitivity analysis, compared with the TAU intervention, the ABA-based interventions continued to have a significant effect, with an increased effect size (SMD = 0.57, 95% CI: 0.28 ~ 0.85, *p* < 0.001). However, the heterogeneity among the studies remained significant, although it decreased slightly according to the sensitivity analysis (*Q* = 19.33, *p* = 0.02, *I*² = 53.57%) (Fig. 7b).

The effect size in the non-TAU group was also significant (SMD = 0.28; 95% CI: 0.07 ~ 0.49; z = 2.57; *p* = 0.01), with nonsignificant heterogeneity (*Q* = 4.24, *p* = 0.64, *I*^2^ < 1%). After the sensitivity analysis was conducted, an increased effect size was detected (SMD = 0.34; 95% CI: 0.10 ~ 0.58; z = 2.78; *p* = 0.01), with nonsignificant heterogeneity (*Q* = 3.03, *p* = 0.70, *I*^2^ < 1%).

**Fig. 8:**
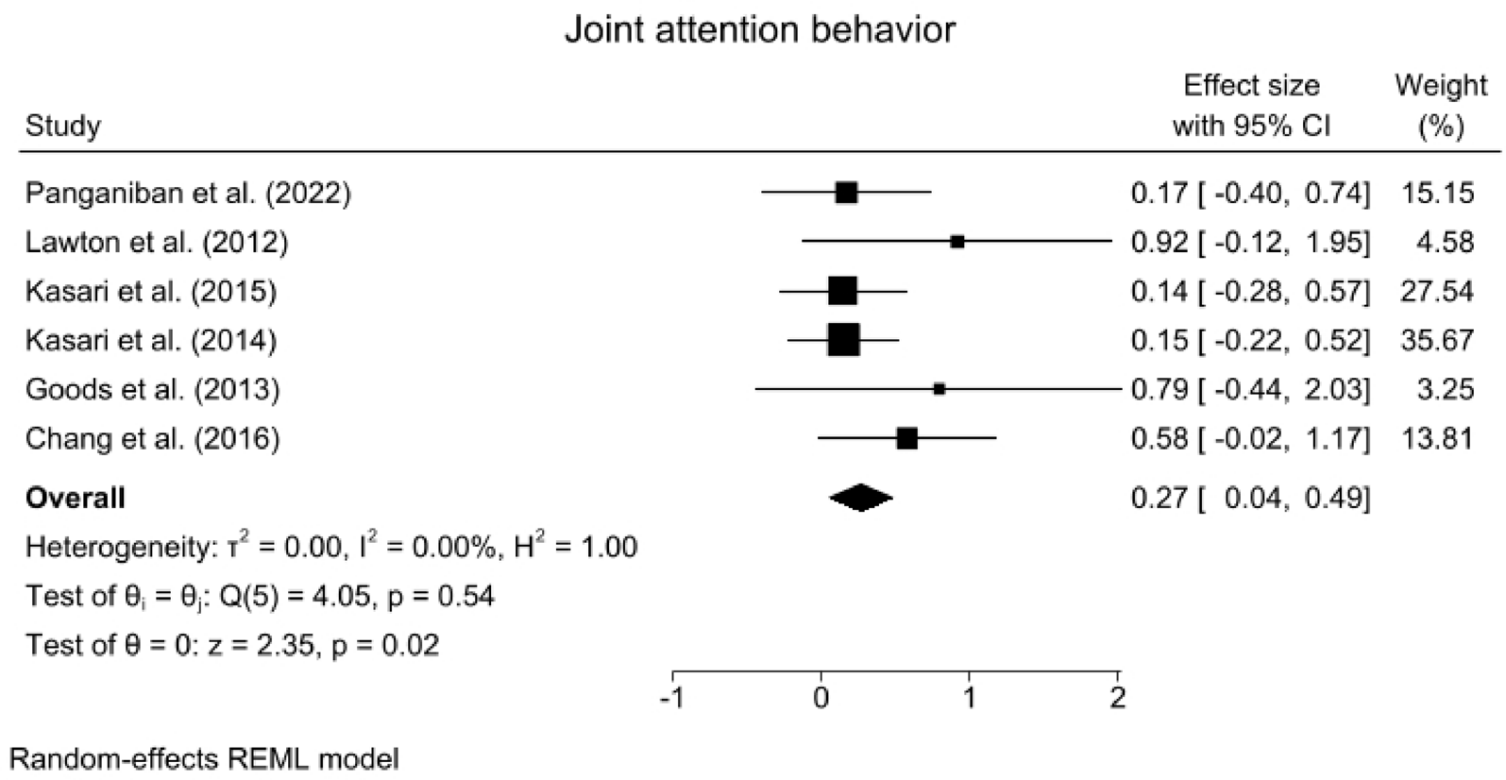
Forest plots for joint attention behavior. *CI* confidence Interval

#### Joint attention behavior

A random effects meta-analysis revealed that compared with the control interventions, the ABA-based interventions significantly affected joint attention behavior (SMD = 0.27, 95% CI: 0.04 ~ 0.49, z = 2.35, *p* = 0.02). Heterogeneity was nonsignificant and small in magnitude (*Q* = 4.05, *p* = 0.54, *I*^2^ < 1%, GRADE = low) (Fig. 8).

A subgroup analysis of treatment intensity and TAU for joint attention behavior was not performed because none of the subgroups included the minimum required number of three studies.

**Fig. 9:**
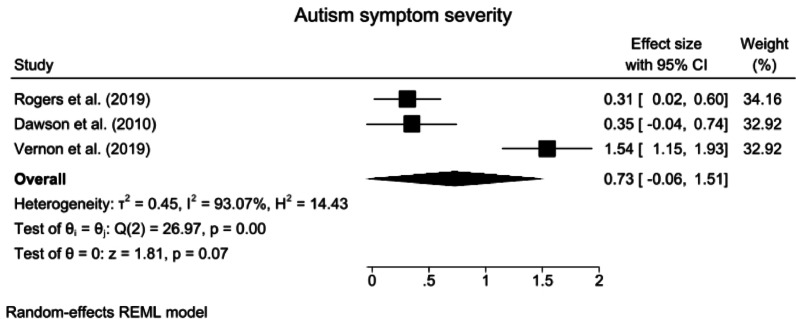
Forest plots for autism symptom severity. *CI* confidence interval

#### Autism symptom severity

The results from a random effects meta-analysis revealed that compared with the control interventions, ABA-based interventions had no effect on the severity of autism symptoms (SMD = 0.73, 95% CI: −0.06 to 1.51, z = 1.81, *p* = 0.07). The heterogeneity was significant and large in magnitude (*Q* = 26.97, *p* < 0.001, *I*^2^ = 93.07%, GRADE = low) (Fig. 9).

A subgroup analysis of treatment intensity and TAU for autism symptom severity was not performed because none of the subgroups included the minimum required number of three studies.

### Results of the qualitative review

The results of this systematic review synthesize the findings from qualitative and mixed-method studies that investigated the implementation, experiences, and outcomes of ABA-based interventions across diverse populations and settings.

### Meta-synthesis of aggregated qualitative studies

Following a categorization analysis, we identified recurring themes across stakeholder perspectives—particularly those of parents and practitioners—regarding the implementation of ABA-based interventions (Table 4). These themes were organized into categories and synthesized into overarching findings. Ultimately, the meaning units extracted from nine studies were classified into ten categories, which were then consolidated into four key synthesized findings: perceived impact, caregiver burden, systemic barriers, and stakeholder advocacy. Together, these syntheses reflect the complex realities and critical tensions that shape the effectiveness and accessibility of ABA interventions in real-world settings.

### Synthesis finding 1 Perceived impact and practical utility of ABA

Across the reviewed studies, caregivers widely perceived ABA as a powerful intervention that generated meaningful improvements in children’s behavior, communication, and socialization. Practical benefits—such as increased verbal expression, compliance with routines, and engagement with peers—led to increased confidence in the utility of ABA, especially when progress became visible. Nevertheless, some parents maintained critical awareness of its limits, cautioning against inflated expectations.

### Category 1.1 Observable behavioral progress in children

Parents described tangible changes in their children’s abilities, particularly in expressive language and social functioning. These gains were directly attributed to the ABA intervention and marked as milestones in the caregiving process.

*My child is now able to verbally express their needs.*

(Park_2016)

*My child is now talking much more than before.*

(Anderson_2017)

*Our child has made real progress since starting ABA.*

*She can now make choices*,* follow simple instructions*,* and even play alongside peers.*

(Grindle et al., 2009)

### Category 1.2 Positive reappraisal of ABA effectiveness

Experiences of child improvement often led to a retrospective reevaluation of the value of ABA. Parents initially hesitant about its structured nature later became vocal proponents, expressing appreciation for the structure and clarity that ABA brought to their caregiving.

*I was skeptical at first*,* but later became a strong advocate.*

(McPhilamy_2013)

*Through ABA*,* I developed a positive view that change is possible.*

(Bang_2021)

*I wish we had known about this kind of programme earlier.*

*It could have saved us a lot of heartache.*

(Grindle et al., 2009)

### Category 1.3 Reservations about effectiveness and expectations

Some families reported that although ABA improved, it did not achieve full recovery or independence for their children. There were concerns about limited comparative advantage over traditional approaches and a continuing need for specialized services.

*No participants achieved full recovery*,* and ongoing special support was necessary.*

*The behavioral outcomes were not significantly different from those of traditional services.*

(Boyd_2001)

### Synthesis finding 2 Burden, exhaustion, and role conflicts in implementation

The implementation of ABA often comes at a psychological and logistical cost to parents. Many reported role strain from serving as both caregivers and interventionists and facing burnout from the continuous demands of home-based programs. In particular, the emotional toll of maintaining high behavioral standards while nurturing the parent‒child relationship was frequently cited.

### Category 2.1 Overwhelming responsibilities and emotional fatigue

Parents expressed feeling physically and mentally overwhelmed by the complexity of ABA implementation. This included coordinating team members, managing session data, and maintaining household responsibilities—often with minimal support.

*I had to be both a mother and a therapist.*

(Park_2016)

*I was exhausted from trying to coordinate everything.*

(Tzanakaki_2012)

### Category 2.2 Internal tension between caregiving and control

Rather than parents, professional therapists working in institutional ABA settings described the internal conflicts they experienced in practice. Some practitioners expressed ethical unease when reinforcement techniques were too close to punishment, blurring the line between behavioral control and child-centered care. This tension reveals how staff can experience value dissonance even when empirically validated protocols are applied.

*Sometimes*,* I felt like I was punishing the children.*

(Bang_2021)

### Synthesis finding 3 Institutional barriers and resource constraints

While individual commitment was high, the systemic infrastructure to support ABA remained weak. Parents repeatedly pointed to inconsistent program delivery, a lack of trained staff, insufficient funding, and minimal policy support. These factors constrain the scope, sustainability, and equity of ABA access.

### Category 3.1 Limited funding and poor governmental support parents frequently

Insufficient public funding was a recurring theme. Parents noted that without proper governmental backing, programs were difficult to maintain and often relied heavily on personal financial investment.

*There is not enough funding to run the program professionally.*

*The degree of government support is minimal.*

(Bang_2021)

### Category 3.2 Shortage of trained staff and program discontinuity

A lack of skilled personnel often leads to interruptions in services. Families expressed concern over staff turnover, inadequate training, and the resulting inconsistencies in care delivery—factors that significantly undermined program efficacy.

*Many professionals were neither properly trained nor evaluated.*

(Dillenburger_2012)

*There were times when the program had to stop due to staff shortages.*

*It is difficult to find ABA-trained staff*,* and the low pay makes it difficult to retain them.*

(Bang_2021)

*Some professionals still do not understand ABA*,* which makes it difficult to obtain support in schools.*

(Grindle et al., 2009)

### Category 3.3 Need for public support and equitable access

Despite positive experiences with ABA, participants voiced strong concern over the lack of public provision. They emphasized that ABA benefits should not be limited to families with the means to pay privately and called for policy change to ensure wider accessibility.

*Parents were satisfied with the ABA but criticized the lack of public funding and support.*

*Public funding is essential to make ABA accessible beyond the private sector.*

(McPhilamy_2013)

### Synthesis finding 4 Ethical identity and advocacy among stakeholders

Across multiple studies, both caregivers and service providers emerged as central figures navigating the ethical terrain of ABA delivery. Parents frequently develop a strong sense of identity as advocates for their children, often challenging institutional barriers to securing appropriate services. On the other hand, front-line staff working in ABA programs described struggles in navigating their professional autonomy, especially when their clinical judgments were constrained by organizational policies or a lack of recognition from other professionals. This synthesis highlights how both groups—although positioned differently—engaged in value-driven action amid systemic resistance.

### Category 4.1 Strong parental/staff advocacy and resilience

Parents often evolve into de facto advocates, not only defending their intervention choices but also actively educating others and pushing them for system-level recognition of ABA. Their testimonies reflect a transformation from passive recipients of care to proactive agents that shape their children’s developmental trajectories.

*I became the advocate my child needed.*

(Park_2016)

*We constantly had to explain and defend our choices.*

(Tzanakaki_2012)

*I believed that ABA would give my child a chance.*

(Anderson_2017)

### Category 4.2 Frustration from lack of authority and autonomy

Front-line practitioners described the emotional and professional strain of having their clinical opinions dismissed or overridden by other institutional actors, such as teachers or administrators. Despite their specialized training and experience with ABA, they reported a lack of decision-making power, contributing to role conflict and disillusionment with systemic structures.

*Even when we wanted to implement ABA*,* our opinions were often dismissed.*

(Bang_2021)

## Discussion

To our knowledge, this study is the first mixed-methods systematic review and meta-analysis to synthesize both the quantitative effectiveness and the qualitative experiences of caregivers and practitioners using ABA-based interventions for children and adolescents with ASD. While the meta-analysis revealed significant improvements in key developmental areas such as adaptive behavior, language, and socialization, it also highlighted areas where ABA-based interventions are less effective, particularly in addressing core areas such as autism symptom severity. Importantly, the methodological rigor of many studies included in our meta-analysis was limited, which introduces a potential risk of bias in the findings.

Moreover, several studies have raised concerns that standardized quantitative measures commonly used in ABA-based intervention research—such as the VABS, SRS, and ADOS—may not fully capture improvements in real-world social functioning [102–104]. For example, observational and caregiver-reported evidence suggests that while children may demonstrate gains during structured assessments, these improvements do not always generalize to unstructured, everyday interactions. However, it is also important to recognize that parent-reported instruments such as the VABS can sometimes overestimate outcomes, introducing another source of bias. This dual possibility implies that the results may be either underestimated or overestimated depending on the measurement context. Therefore, we emphasize that the results must be interpreted with caution.

### Quantitative synthesis

The meta-analysis indicated that ABA-based interventions resulted in significant improvements in adaptive behavior, daily living skills, socialization, joint attention, and language, findings that generally align with prior reviews reporting benefits in functional and developmental domains [24, 25, 27]. Language outcomes were particularly responsive, with high-intensity interventions showing stronger effects, which is consistent with dose–response findings in the literature [24, 27]. Sensitivity analyses further confirmed the robustness of these effects, although variability across studies highlights ongoing challenges in generalizability.

However, other studies have reported nonsignificant results in adaptive behavior and daily living skills [28, 105, 106], likely reflecting differences in intervention models, outcome selection, and methodological rigor. In our comparative analyses, ABA-based interventions showed advantages over TAU for adaptive and daily living skills; however, effects on socialization were not statistically significant, and interpretation should take into account the heterogeneity of TAU. In this review, TAU was highly heterogeneous, incorporating multiple clinical and community-based approaches, as outlined in the *Methods* section. Such multimodal and family-centered elements likely contributed both to the absence of a consistent advantage for socialization [61, 65, 75], and to between-study variability in effect sizes. Moreover, several widely used standardized measures of socialization—such as the ADOS–II and the SRS–II—provide only partial ecological coverage of spontaneous, peer-to-peer interactions, helping to explain discrepancies with caregiver observations in daily life [107–109]. Consistent with this view, recent work has highlighted the need for more naturalistic outcome tools—e.g., structured peer-observation scales developed for real-world classrooms such as the Autism Peer Interaction Observation Scale (APIOS)—and in-situ approaches like Ecological Momentary Assessment (EMA) to better capture everyday social exchanges [110, 111].

On the other hand, several commonly used measures in these domains are collected under procedures designed to limit expectancy bias—for example, the ADOS is clinician-administered and often scored by blinded assessors in trials—so the nonsignificant socialization effects may also reflect genuine constraints in targeting core autistic social features [59, 99]. By contrast, reporter-based instruments such as the SRS–II [88], and interview-/report-based indices such as the VABS–I and VABS–II [85, 86], can be more sensitive to rater and context effects and may overestimate outcomes. Taken together, these considerations indicate that estimates can be pulled in both directions—by potential undercapture of everyday peer interactions and by potential overestimation in reporter-based tools—underscoring the need for cautious interpretation and for future trials to specify, measure, and stratify both TAU content/intensity and the ecological validity of outcome measures [112–114].

Meanwhile, no significant effects emerged for autism symptom severity, a finding consistent with those of earlier reviews [22]. ABA-based interventions may not significantly reduce core autism symptom severity for several reasons. First, the duration and intensity of most protocols (typically 6–12 months) may be insufficient to influence deeply rooted neurodevelopmental features, whereas adaptive skills and language skills often respond more readily to intensively delivered, short-duration programs [14]. Second, gains achieved in structured sessions may not generalize or be maintained in naturalistic contexts such as home or school [15]. Third, the heterogeneity of ASD—spanning wide variations in cognitive, communicative, and behavioral profiles—dilutes average treatment effects, making group-level changes difficult to detect [115]. Finally, improvements in joint attention were observed, but given the small number of eligible studies, further high-quality research is needed to substantiate these results and examine long-term outcomes.

### Qualitative synthesis

This qualitative synthesis reveals that ABA-based interventions are perceived by stakeholders—particularly parents and practitioners—as both highly transformative and significantly demanding. The findings illuminate four central domains: perceived impact, caregiver burden, systemic barriers, and stakeholder advocacy. Together, they illustrate the multifaceted reality of implementing ABA in real-world settings and the critical tensions that shape its effectiveness and accessibility.

Many parents reported significant improvements in their children’s behavior, language, and social functioning after engaging in ABA. These tangible outcomes validated for the method’s clinical utility and encouraged long-term engagement. Particularly noteworthy were instances in which previously nonverbal children began to express their needs or interact with peers—clear indicators of meaningful developmental progress. However, the findings also revealed that ABA does not uniformly result in full functional recovery. This gap between progress and cure highlights the importance of setting realistic expectations during program initiation, especially in clinical and educational counseling [14, 19]. Overstating ABA’s potential may lead to caregiver disillusion or withdrawal from essential complementary services. Future program designs should ensure clear communication regarding expected outcomes, the variability of progress, and the continuing need for support.

The burden of home-based ABA implementation has emerged as a significant source of emotional and physical exhaustion, particularly for mothers [116]. As the dual role of parent and therapist blurred boundaries, many caregivers reported identity strain, fatigue, and isolation [117]. These findings underscore a structural issue: owing to the scarcity of BCBAs and trained professionals—especially in underserved regions—telehealth and home-based ABA models have often been devolved into a default solution [118], placing disproportionate responsibility on families.

Rather than being supported by adequate professional oversight, parents are frequently left to coordinate complex behavioral programs with minimal guidance [82]. While home-based delivery is sometimes framed as empowering, the evidence suggests that it is often an unintended consequence of systemic inadequacies. Programs should incorporate systemic caregiver support—including supervision, respite care, and peer networks—to sustain long-term implementation and mitigate psychological risk [119, 120]. Additionally, shifting responsibilities to parents should not be regarded merely as cost-saving but should be evaluated through ethical and clinical lenses that prioritize caregiver well-being.

While ABA relies on standardized protocols, this review highlights how front-line staff sometimes experience moral discomfort when procedures feel excessively controlling or punitive. The reported internal conflict—“feeling like punishing children”—suggests that ethical training and reflective supervision must constitute integral components of practitioner development [78]. Importantly, many of the qualitative accounts captured in our synthesis may reflect practice climates predating recent professional updates; current behavior-analytic ethics and practice guidance explicitly emphasize a compassionate, dignity-preserving, assent-sensitive, and least-restrictive approach, thereby providing clearer safeguards against procedures that could be experienced as coercive [121]. In this context, reflective supervision and practitioner training are not only recommended but are increasingly structured to operationalize these principles—e.g., strengthening assent-based planning, prioritizing reinforcement-based supports, and formalizing review/feedback loops—so that earlier sources of discomfort are progressively mitigated in contemporary settings [121–123].

Despite personal commitment from both caregivers and staff, structural inadequacies—namely, insufficient public funding, workforce instability, and policy ambivalence—were recurrent themes [124]. The shortage of trained professionals not only disrupted program continuity but also placed additional burdens on caregivers, leading to program dropout or suboptimal fidelity. Notably, parental satisfaction with ABA was frequently accompanied by frustration over its exclusivity to those with private means. This duality underscores a pressing equity issue: if ABA is to be considered a gold-standard intervention, its availability should not be contingent upon socioeconomic status [124]. Governments and health systems must invest in sustainable workforce development, subsidy models, and public education to ensure equitable access and quality assurance.

Both parents and practitioners emerged as advocates within systems that often fail to recognize their expertise [125, 126]. Parents fought for services and legitimacy, whereas therapists struggled with limited authority to apply evidence-based practices in institutional settings. These findings point to pervasive power asymmetries in multidisciplinary environments, where ABA knowledge is undervalued or siloed. Cross-sector training, greater inclusion of ABA specialists in IEP and policy decisions, and clearer role delineation may help mitigate these tensions. Ultimately, service models must move beyond technical delivery to recognize and empower those at the frontline of care.

### Implications for healthcare policy and clinical practice

This review highlights several actionable implications for clinical practice and healthcare policy. In the quantitative results, a dose–response effect was observed only in language outcomes, while ABA-based interventions showed advantages over TAU in adaptive behavior, language, and daily living skills. In the qualitative results, it was reported that when parents assumed an excessive therapeutic role, fatigue and reduced fidelity accumulated. Taken together, these findings suggest that the effectiveness of ABA-based interventions cannot be explained solely by quantitative factors such as session number or duration, but rather depends on goal specification, implementation structure, and the distribution of responsibility. This indicates that even when sufficient intensity is secured, effectiveness may be constrained if delivery is fragmented or excessively reliant on families.

Professionally led models ensure procedural fidelity and accelerate target attainment, but they are resource-intensive and may lack sufficient generalization across home and school contexts. Conversely, parent-mediated or hybrid models are advantageous for everyday generalization, yet when structural supports are absent and responsibility is disproportionately shifted to parents, burden and inequities are exacerbated. The qualitative synthesis highlighted the “blurring of boundaries between parent and therapist roles,” which clearly illustrates this point. Therefore, future intervention designs should systematize parent training and coaching, incorporate respite and peer supports, and avoid expanding parent-mediated models merely on the basis of cost reduction.

Family quality of life (QoL) and functional well-being were not included in the meta-analysis, as heterogeneity of instruments and inconsistent reporting rendered synthesis unfeasible. However, parental burden and family-level effects were repeatedly confirmed in the qualitative synthesis, demonstrating that QoL must be treated as a core outcome in clinical and policy decision-making. Future research should designate QoL, caregiver burden, child participation, and daily functioning as minimum core outcomes, measured at fixed intervals. These should not be regarded as supplementary indicators but as essential elements for verifying the real value of interventions.

From the standpoint of generalizability, the most salient limitation is the overwhelming concentration of studies in high-income countries. Pervin et al. reported that studies from low- and middle-income countries (LMICs) account for less than 10% of the total ASD intervention literature, and are of insufficient quality to allow meaningful comparison [127]. Durkin et al. further emphasized that more than 80% of ASD research has been conducted in North America, Europe, and Japan, while LMIC contexts, which encompass the majority of the world’s children, have been effectively excluded [128]. In Jordan, cost and transportation were the most frequently reported barriers to service access [129], while in Ethiopia there was an absence of culturally appropriate diagnostic tools [130]. These imbalances not only bias the research evidence but also create profound inequities in access to services. In such settings, community health worker involvement, school-based group programs, and modular ABA/NDBI models with tele-coaching have been proposed as realistic alternatives.

Cultural norms also shape caregiver roles and expectations, significantly influencing intervention processes. A comparative study of the United Kingdom and China reported that while professional delegation is more common in the UK, Chinese families, influenced by Confucian values, more frequently adopt a mother-centered, parent-as-therapist model [131]. Ferretti et al. found that in multicultural families in the United States, language barriers and cultural discomfort with having outsiders in private family spaces hindered parent–provider relationship-building [132]. These findings indicate that intervention effects derived from single cultural contexts cannot be readily generalized, and that service design must explicitly incorporate culturally and linguistically responsive strategies for role negotiation and trust-building.

Institutional and insurance contexts also directly shape real-world effectiveness. In the United States, state-level insurance mandates have established ABA as a medically necessary service [133, 134]. In Europe, however, government endorsement has been limited [135], while in Nordic countries educational models such as TEACCH remain dominant [136]. In Canada, support varies by province, and in Australia ABA is one of several options under the national disability insurance scheme, alongside developmental approaches. These differences extend beyond policy choices to affect workforce supply, access costs, and the lived experiences of families.

In conclusion, the existing evidence remains heavily skewed toward high-income contexts, and generalization without consideration of national institutional and cultural factors has clear limitations. Future research should expand multi-regional and multicultural inclusion, report intervention intensity and implementation structure with greater precision, and designate QoL and functional outcomes as core evaluation domains. Only then can ABA-based interventions establish genuinely generalizable evidence that contributes to the lives of children with autism and their families.

The findings highlight that ABA-based interventions should be integrated into individualized care plans with attention to intensity, delivery structure, and family burden. Policymakers need to expand workforce training and public funding to address provider shortages and inequities, while embedding quality-of-life and caregiver outcomes as benchmarks for evaluation. Cross-national disparities further require scalable models suitable for low-resource contexts and culturally responsive strategies that align interventions with diverse family expectations and institutional systems.

### Limitations and strengths of this review

This study has several limitations that warrant consideration. First, while our focus on RCTs ensured high reliability of evidence, it excluded valuable insights from single-case design (SCD) studies, which are particularly relevant to the individualized approach of ABA-based interventions [34]. Future research should integrate findings from RCTs, observational studies, and SCDs to provide a more holistic understanding of the effectiveness of ABA-based interventions.

Second, the heterogeneity observed in some meta-analyses highlights the need for further exploration of the moderators that influence treatment outcomes. Factors such as demographic characteristics, intervention settings, and practitioners’ expertise may play significant roles in determining the success of ABA-based interventions. Third, while subgroup analyses were conducted for intervention intensity and TAU, additional analyses based on participants’ demographics, caregivers’ involvement, and intervention features would provide more nuanced insights. Fourth, comorbidities were not addressed in this review, despite their high prevalence and potential influence on treatment outcomes in children with ASD. The presence of co-occurring conditions—such as ADHD, anxiety, or intellectual disability—may alter response patterns, moderate effectiveness, or impact the generalizability of findings. Future research should systematically examine how comorbidities interact with intervention processes and outcomes to inform more individualized and clinically realistic applications. Finally, the qualitative synthesis was limited by the small number of studies and the potential reporting biases inherent in qualitative research.

Despite these limitations, this review has several key strengths. The use of a mixed-methods approach provides a comprehensive assessment of both quantitative outcomes and qualitative experiences to address a significant gap in the literature effectively [137]. Moreover, the inclusion of sensitivity analyses and subgroup comparisons further strengthens the reliability and robustness of the findings. To avoid the potential for overreporting of results, combined SMD and SE were utilized to ensure more precise and dependable conclusions. Furthermore, this review broadened the scope of the analysis by incorporating a wider array of intervention types and outcome measures than those explored in prior studies do, which offers a more complete understanding of the impact of ABA-based interventions.

Unlike earlier meta-analyses that used a fixed-effect model or unstandardized calculations [23, 138], this review employed a random-effects model with standardized estimates, which provides more reliable and generalizable results. Additionally, a comparison with TAU was conducted to provide a clearer demonstration of the therapeutic effects of ABA-based interventions and to offer stronger evidence of its effectiveness compared with standard treatment practices.

## Conclusion

This mixed-methods systematic review and meta-analysis provides an integrated synthesis of the current evidence on ABA-based interventions for children and adolescents with ASD. While the findings suggest potential benefits for developmental and behavioral outcomes, these results must be interpreted with caution. Most included studies carried a high risk of bias, and the GRADE assessment rated the quality of evidence as moderate for language skills, very low for autism symptom severity, and low for other outcomes, with notable heterogeneity particularly in language outcomes. By combining quantitative and qualitative perspectives, this study not only highlights possible areas of improvement but also highlights the substantial challenges faced by caregivers and practitioners. Future research should adopt more rigorous and inclusive methodologies and systematically examine moderators to better clarify the effectiveness and accessibility of ABA-based interventions across diverse populations.

## Supplementary Information

Supplementary Material 1.

## Data Availability

The data are presented in the manuscript and supplementary materials. The raw data used for the meta-analyses are available from the corresponding author on reasonable request.
