# Supplementary material for: Effectiveness and experiences of early intensive behavioral and naturalistic developmental behavior interventions for autism spectrum disorders: a mixed-methods systematic review and meta-analysis
Source: Child Adolesc Psychiatry Ment Health. 2025 Dec 26;20:14. doi: 10.1186/s13034-025-00997-z (PMC12849440; doi:10.1186/s13034-025-00997-z)
Supplement: Supplementary file 1 — Supplementary Material 1. [file 13034_2025_997_MOESM1_ESM.docx]

**Supplementary Methods**

This study included several cases where a single outcome was measured via multiple assessments. Treating these as separate outcomes posed the risk of overestimating variance. To mitigate this risk, the mean effect size and combined standard error (SE) were calculated via formulas proposed by Borenstein and colleagues (1). The following formulas were applied:

1. Estimated variance for studies reporting two outcomes.

$$V_{\bar{Y}}=1/4\left( V_{Y1}+V_{Y2}+2r\sqrt{V_{Y1}}\sqrt{V_{Y2}} \right)$$

1. Estimated variance for studies reporting more than three outcomes.

$$V_{\bar{Y}}=\left( \frac{1}{m} \right)^{2}var\left( \sum_{i=1}^{m} Yi \right)=\left( \frac{1}{m} \right)^{2}\left( \sum_{i=1}^{m} Yi+\sum_{i\neq k} \left( r_{ik}\sqrt{V_{i}}\sqrt{V_{k}} \right) \right)$$

(1) Borenstein M, Hedges LV, Higgins JP, Rothstein HR. Introduction to meta-analysis: John Wiley & Sons; 2021.

**eTable 1. Applied keywords and the search results from each database.**

| Database | # | Search terms | *n* |
| --- | --- | --- | --- |
| OVID-  Medline  (8.3.2023.) | 1 | exp Child Development Disorders, Pervasive/ | 46537 |
|  | 2 | pervasive development* disorder*.mp | 2154 |
|  | 3 | autis*.mp | 67756 |
|  | 4 | Asperger*.mp | 2899 |
|  | 5 | kanner*.mp | 272 |
|  | 6 | childhood schizophrenia.mp | 279 |
|  | 7 | develop* delay.mp | 16364 |
|  | 8 | social communication disorder*.mp | 189 |
|  | 9 | (PDD or PDDs or ASD or ASDs or SCD).mp | 54174 |
|  | 10 | or/1-9 | 112350 |
|  | 11 | Early Intervention, Educational/ | 3485 |
|  | 12 | Behavior Therapy/ | 30116 |
|  | 13 | Applied Behavior Analysis/ | 108 |
|  | 14 | applied behavio* analy*.mp | 986 |
|  | 15 | ABA.mp | 16030 |
|  | 16 | Lovaas*.mp | 44 |
|  | 17 | (intensive adj3 (interven* or therap* or treat* or program*)).mp | 40159 |
|  | 18 | (EIBI or IBI).mp | 1043 |
|  | 19 | (naturalistic adj3 (interven* or therap* or treat* or program*)).mp | 881 |
|  | 20 | (NDBI or NDBIs).mp | 6 |
|  | 21 | discrete trial teaching.mp | 48 |
|  | 22 | DTT.mp | 6150 |
|  | 23 | picture exchange communication system.mp | 97 |
|  | 24 | PECS.mp | 1988 |
|  | 25 | early start denver model.mp | 92 |
|  | 26 | ESDM.mp | 83 |
|  | 27 | pivotal response treatment.mp | 59 |
|  | 28 | PRT.mp | 2091 |
|  | 29 | (joint attention and symbolic play).mp. | 35 |
|  | 30 | (JASPER or JASP).mp | 363 |
|  | 31 | or/11-30 | 101746 |
|  | 32 | **10 and 31** | **2877** |
| OVID-  EMbase  (8.3.2023.) | 1 | exp autism/ | 92479 |
|  | 2 | pervasive development* disorder*.mp | 4008 |
|  | 3 | autis*.mp | 100464 |
|  | 4 | Asperger*.mp | 6054 |
|  | 5 | kanner*.mp | 383 |
|  | 6 | childhood schizophrenia.mp | 206 |
|  | 7 | develop* delay.mp | 32777 |
|  | 8 | social communication disorder*.mp | 172 |
|  | 9 | (PDD or PDDs or ASD or ASDs or SCD).mp | 86708 |
|  | 10 | or/1-9 | 186884 |
|  | 11 | early childhood intervention/ | 3140 |
|  | 12 | behavior therapy/ | 45341 |
|  | 13 | behavior modification/ | 8499 |
|  | 14 | applied behavio* analy*.mp | 901 |
|  | 15 | ABA.mp | 15499 |
|  | 16 | Lovaas*.mp | 53 |
|  | 17 | (intensive adj3 (interven* or therap* or treat* or program*)).mp | 61753 |
|  | 18 | (EIBI or IBI).mp | 1678 |
|  | 19 | (naturalistic adj3 (interven* or therap* or treat* or program*)).mp | 1177 |
|  | 20 | (NDBI or NDBIs).mp | 8 |
|  | 21 | discrete trial teaching.mp | 49 |
|  | 22 | DTT.mp | 7973 |
|  | 23 | picture exchange communication system.mp | 138 |
|  | 24 | PECS.mp | 2781 |
|  | 25 | early start denver model.mp | 140 |
|  | 26 | ESDM.mp | 130 |
|  | 27 | pivotal response treatment.mp | 82 |
|  | 28 | PRT.mp | 3651 |
|  | 29 | (joint attention and symbolic play).mp. | 47 |
|  | 30 | (JASPER or JASP).mp | 653 |
|  | 31 | or/11-30 | 150302 |
|  | 32 | **10 and 31** | **4073** |
| CENTRAL  (8.3.2023.) | 1 | exp Child Development Disorders, Pervasive/ | 2391 |
|  | 2 | “pervasive developmental disorder” OR “pervasive developmental disorders” | 272 |
|  | 3 | autis* | 4841 |
|  | 4 | Asperger* | 283 |
|  | 5 | kanner* | 7 |
|  | 6 | “childhood schizophrenia” | 9 |
|  | 7 | “developmental delay” | 476 |
|  | 8 | “social communication disorder” OR “social communication disorders” | 21 |
|  | 9 | PDD OR PDDs OR ASD OR ASDs OR SCD | 4732 |
|  | 10 | or/1-9 | 7911 |
|  | 11 | Early Intervention, Educational/ | 607 |
|  | 12 | Behavior Therapy/ | 5424 |
|  | 13 | Applied Behavior Analysis/ | 8 |
|  | 14 | “applied behavior analysis” OR “applied behaviour analysis” | 90 |
|  | 15 | ABA | 462 |
|  | 16 | Lovaas* | 7 |
|  | 17 | intensive NEAR/3 (interven* OR therap* OR treat* OR program*) | 12098 |
|  | 18 | EIBI OR IBI | 121 |
|  | 19 | naturalistic NEAR/3 (interven* OR therap* OR treat* OR program*) | 195 |
|  | 20 | NDBI OR NDBIs | 0 |
|  | 21 | “discrete trial teaching” | 11 |
|  | 22 | DTT | 91 |
|  | 23 | “picture exchange communication system” | 24 |
|  | 24 | PECS | 366 |
|  | 25 | “early start denver model” | 50 |
|  | 26 | ESDM | 51 |
|  | 27 | “pivotal response treatment” | 37 |
|  | 28 | PRT | 612 |
|  | 29 | “joint attention” AND “symbolic play” | 26 |
|  | 30 | JASPER OR JASP | 81 |
|  | 31 | or/11-30 | 19719 |
|  | 32 | **10 and 31** | **512**  **(CENTRAL: 505)** |
| Ovid APA PsycInfo  (8.3.2023.) | 1 | exp Autism Spectrum Disorders/ | 52998 |
|  | 2 | pervasive development* disorder*.mp | 3356 |
|  | 3 | autis*.mp | 67842 |
|  | 4 | Asperger*.mp | 4686 |
|  | 5 | kanner*.mp | 526 |
|  | 6 | childhood schizophrenia.mp | 619 |
|  | 7 | develop* delay.mp | 3596 |
|  | 8 | social communication disorder*.mp | 193 |
|  | 9 | (PDD or PDDs or ASD or ASDs or SCD).mp | 28916 |
|  | 10 | or/1-9 | 75316 |
|  | 11 | Early Intervention/ | 12329 |
|  | 12 | Behavior Modification/ | 10714 |
|  | 13 | Behavior Therapy/ | 15755 |
|  | 14 | Applied Behavior Analysis/ | 1264 |
|  | 15 | applied behavio* analy*.mp | 2929 |
|  | 16 | ABA.mp | 1838 |
|  | 17 | Lovaas*.mp | 167 |
|  | 18 | (intensive adj3 (interven* or therap* or treat* or program*)).mp | 9506 |
|  | 19 | (EIBI or IBI).mp | 459 |
|  | 20 | (naturalistic adj3 (interven* or therap* or treat* or program*)).mp | 1044 |
|  | 21 | (NDBI or NDBIs).mp | 5 |
|  | 22 | discrete trial teaching.mp | 152 |
|  | 23 | DTT.mp | 345 |
|  | 24 | picture exchange communication system.mp | 230 |
|  | 25 | PECS.mp | 281 |
|  | 26 | early start denver model.mp | 114 |
|  | 27 | ESDM.mp | 96 |
|  | 28 | pivotal response treatment.mp | 129 |
|  | 29 | PRT.mp | 439 |
|  | 30 | (joint attention and symbolic play).mp. | 61 |
|  | 31 | (JASPER or JASP).mp | 305 |
|  | 32 | or/11-31 | 52650 |
|  | 33 | **10 and 32** | **5337** |
| EBSCOhost CINAHL Ultimate  (8.3.2023.) | 1 | (MH "Child Development Disorders, Pervasive+") | 31634 |
|  | 2 | TI pervasive development* disorder* OR AB pervasive development* disorder* | 863 |
|  | 3 | TI autis* OR AB autis* | 32558 |
|  | 4 | TI Asperger* OR AB Asperger* | 1565 |
|  | 5 | TI kanner* OR AB kanner* | 49 |
|  | 6 | TI childhood schizophrenia OR AB childhood schizophrenia | 290 |
|  | 7 | TI develop* delay OR AB develop* delay | 6249 |
|  | 8 | TI social communication disorder* OR AB social communication disorder* | 444 |
|  | 9 | TI ( (PDD or PDDs or ASD or ASDs or SCD) ) OR AB ( (PDD or PDDs or ASD or ASDs or SCD) ) | 18799 |
|  | 10 | S1 OR S2 OR S3 OR S4 OR S5 OR S6 OR S7 OR S8 OR S9 | 52420 |
|  | 11 | (MH "Early Childhood Intervention") | 6275 |
|  | 12 | (MH "Behavior Modification") | 2751 |
|  | 13 | (MH "Behavior Therapy") | 13570 |
|  | 14 | (MH "Applied Behavior Analysis") | 280 |
|  | 15 | TI applied behavio* analy* OR AB applied behavio* analy* | 544 |
|  | 16 | TI ABA OR AB ABA | 1036 |
|  | 17 | TI Lovaas* OR AB Lovaas* | 24 |
|  | 18 | TI ( intensive W3 (interven* or therap* or treat* or program*) ) OR AB ( intensive W3 (interven* or therap* or treat* or program*) ) | 12122 |
|  | 19 | TI ( EIBI or IBI ) OR AB ( EIBI or IBI ) | 336 |
|  | 20 | TI ( naturalistic W3 (interven* or therap* or treat* or program*) ) OR AB ( naturalistic W3 (interven* or therap* or treat* or program*) ) | 320 |
|  | 21 | TI ( NDBI or NDBIs ) OR AB ( NDBI or NDBIs ) | 0 |
|  | 22 | TI discrete trial teaching OR AB discrete trial teaching | 43 |
|  | 23 | TI DTT OR AB DTT | 360 |
|  | 24 | TI picture exchange communication system OR AB picture exchange communication system | 95 |
|  | 25 | TI PECS OR AB PECS | 586 |
|  | 26 | TI early start denver model OR AB early start denver model | 63 |
|  | 27 | TI ESDM OR AB ESDM | 42 |
|  | 28 | TI pivotal response treatment OR AB pivotal response treatment | 55 |
|  | 29 | TI PRT OR AB PRT | 584 |
|  | 30 | TI ( “joint attention” and “symbolic play” ) OR AB ( “joint attention” and “symbolic play” ) | 26 |
|  | 31 | TI ( JASPER or JASP ) OR AB ( JASPER or JASP ) | 195 |
|  | 32 | S11 OR S12 OR S13 OR S14 OR S15 OR S16 OR S17 OR S18 OR S19 OR S20 OR S21 OR S22 OR S23 OR S24 OR S25 OR S26 OR S27 OR S28 OR S29 OR S30 OR S31 | 37689 |
|  | 33 | **S10 AND S32** | **2800** |

| Database | # | Search terms | *n* |
| --- | --- | --- | --- |
| KoreaMed  (2023.03.08.) | 1 | (("applied behavior analysis"[ALL])) OR ("applied behaviour analysis"[ALL]) | 2 |
|  | 2 | ("early intensive behavioral intervention"[ALL]) | 1 |
|  | 3 | ("naturalistic developmental behavioral intervention"[ALL]) | 0 |
|  | 4 | or/1-3 | 13 |
| Kmbase  (2023.03.08.) | 1 | ([ALL=applied behavior analysis] OR [ALL=applied behaviour analysis]) | 13 |
|  | 2 | [ALL=early intensive behavioral intervention] | 3 |
|  | 3 | [ALL=naturalistic developmental behavioral intervention] | 0 |
|  | 4 | ([ALL=응용행동분석] OR [ALL=응용 행동 분석]) | 10 |
|  | 5 | ([ALL=조기중재] OR [ALL=조기 중재]) | 48 |
|  | 6 | ([ALL=조기개입] OR [ALL=조기 개입]) | 36 |
|  | 7 | ([ALL=조기행동] OR [ALL=조기 행동]) | 3 |
|  | 8 | ([ALL=조기집중] OR [ALL=조기 집중]) | 8 |
|  | 9 | (([ALL=자연] AND [ALL=발달]) AND [ALL=행동]) | 13 |
|  | 10 | [ALL=자연적 중재] | 4 |
|  | 11 | [ALL=자연주의 중재] | 0 |
|  | 12 | or/1-11 | 145 |

**eTable 2. List of Excluded Studies and Reasons for Exclusion.**

| < Reasons for Exclusion >  1. Studies not involving the predefined population.  2. Studies not utilizing the predefined intervention.  3. Studies not including the predefined control group.  4. Studies not reporting the predefined medical outcomes.  5. Studies not adhering to the predefined study design. |
| --- |

| No. | Author | Year | Title | Journal | Reason for Exclusion |
| --- | --- | --- | --- | --- | --- |
| 1 | Schreibman | 2014 | A randomized trial comparison of the effects of verbal and pictorial naturalistic communication strategies on spoken language for young children with autism | Journal of Autism and Developmental Disorders | 3 |
| 2 | Fuller | 2019 | A multi-component communication intervention for pre-verbal children with autism: optimizing outcomes | Journal of Intellectual Disability Research | 2 |
| 3 | McClure | 2021 | Connecting the Dots: a cluster-randomized clinical trial integrating standardized autism spectrum disorders screening, high-quality treatment, and long-term outcomes | BMC trials | 5 |
| 4 | Fernell | 2011 | Early intervention in 208 Swedish preschoolers with autism spectrum disorder. A prospective naturalistic study | Research in Developmental Disabilities | 3 |
| 5 | Drapalik | 2022 | Feasibility and Acceptability of Delivering Pivotal Response Treatment for Autism Spectrum Disorder via Telehealth: Pilot Pre-Post Study | JMIR Pediatrics and Parenting | 5 |
| 6 | Weitlauf | 2022 | A Longitudinal RCT of P-ESDM With and Without Parental Mindfulness Based Stress Reduction: Impact on Child Outcomes | Journal of Autism and Developmental Disorders | 2 |
| 7 | Engelstad | 2020 | Early Achievements for Education Settings: An Embedded Teacher-Implemented Social Communication Intervention for Preschoolers With Autism Spectrum Disorder | Perspectives of the ASHA Special Interest Groups | 2 |
| 8 | Rogers | 2019 | Enhancing Low-Intensity Coaching in Parent Implemented Early Start Denver Model Intervention for Early Autism: a Randomized Comparison Treatment Trial | Journal of Autism and Developmental Disorders | 3 |
| 9 | Mohammadzaheri | 2022 | Neural correlates of enhancing question asking and initiations in children with autism spectrum disorders: A Randomized Clinical Trial | Social Neuroscience | 4 |
| 10 | Roberts | 2015 | Early Intervention for Toddlers With Language Delays: A Randomized Controlled Trial | Pediatrics | 2 |
| 11 | Touzet | 2017 | Impact of the Early Start Denver Model on the cognitive level of children with autism spectrum disorder: study protocol for a randomised controlled trial using a two-stage Zelen design | BMJ Open | 5 |
| 12 | Shire | 2022 | Exploring coaching and follow-up supports in community-implemented caregiver-mediated JASPER intervention | Autism | 5 |
| 13 | Mirenda | 2022 | A Randomized, Community-Based Feasibility Trial of Modified ESDM for Toddlers with Suspected Autism | Journal of Autism and Developmental Disorders | 5 |
| 14 | Beaudoin | 2019 | Parent-mediated intervention tends to improve parent-child engagement, and behavioral outcomes of toddlers with ASD-positive screening: a randomized crossover trial | Research in Autism Spectrum Disorders | 5 |
| 15 | Sanders | 2020 | Addressing Challenging Behavior During Hospitalizations for Children with Autism: A Pilot Applied Behavior Analysis Randomized Controlled Trial | Autism Research | 4 |
| 16 | Rogers | 2021 | A Multisite Randomized Controlled Trial Comparing the Effects of Intervention Intensity and Intervention Style on Outcomes for Young Children With Autism | Journal of the American Academy of Child and Adolescent Psychiatry | 3 |
| 17 | Gilroy | 2018 | A pilot community-based randomized comparison of speech generating devices and the picture exchange communication system for children diagnosed with autism spectrum disorder | Autism Research | 3 |
| 18 | Shire | 2020 | Peer engagement in toddlers with autism: Community implementation of dyadic and individual Joint Attention, Symbolic Play, Engagement, and Regulation intervention | Autism | 3 |
| 19 | Stahmer | 2022 | A Waitlist Randomized Implementation Trial of Classroom Pivotal Response Teaching for Students With Autism | Focus on Autism and Other Developmental Disabilities | 5 |
| 20 | Eric | 2020 | The applied behaviour analysis imperative in the management of autism | Swiss Archives of Neurology, Psychiatry and Psychotherapy | 5 |
| 21 | Estes | 2014 | The impact of parent-delivered intervention on parents of very young children with autism | Journal of Autism and Developmental Disorders | 1 |
| 22 | Yoder | 2006 | A randomized comparison of the effect of two prelinguistic communication interventions on the acquisition of spoken communication in preschoolers with ASD | Journal of Speech, Language, and Hearing Research | 3 |
| 23 | Manohar | 2019 | Brief Parent-Mediated Intervention for Children with Autism Spectrum Disorder: a Feasibility Study from South India | Journal of Autism and Developmental Disorders | 5 |
| 24 | Bradshaw | 2019 | The Use of Eye Tracking as a Biomarker of Treatment Outcome in a Pilot Randomized Clinical Trial for Young Children with Autism | Autism Research | 4 |
| 25 | Rivard | 2021 | Using Prevent Teach Reinforce for Young Children to Manage Challenging Behaviors in Public Specialized Early Intervention Services for Autism | Journal of Autism and Developmental Disorders | 2 |
| 26 | DeKorte | 2020 | Self-initiations in young children with autism during Pivotal Response Treatment with and without robot assistance | Autism | 3 |
| 27 | Davis | 2022 | Caregiver responsiveness as a mechanism to improve social communication in toddlers: Secondary analysis of a randomized controlled trial | Autism Research | 4 |
| 28 | Vismara | 2018 | Telehealth Parent Training in the Early Start Denver Model: results From a Randomized Controlled Study | Focus on Autism and Other Developmental Disabilities | 2 |
| 29 | Howlin | 2007 | The effectiveness of Picture Exchange Communication System (PECS) training for teachers of children with autism: A pragmatic, group randomised controlled trial | Journal of Child Psychology and Psychiatry | 5 |
| 30 | Sallows | 2005 | Intensive behavioral treatment for children with autism: Four-year outcome and predictors | American journal on mental retardation | 3 |
| 31 | Smith | 2000 | Randomized trial of intensive early intervention for children with pervasive developmental disorder | American journal on mental retardation | 3 |
| 32 | Shih | 2021 | Joint engagement is a potential mechanism leading to increased initiations of joint attention and downstream effects on language: JASPER early intervention for children with ASD | Journal of Child Psychology and Psychiatry | 4 |
| 33 | Vivanti | 2019 | Outcomes of children receiving Group-Early Start Denver Model in an inclusive versus autism-specific setting: A pilot randomized controlled trial | Autism | 3 |
| 34 | Gordon | 2011 | A communication-based intervention for nonverbal children with autism: what changes? Who benefits? | Journal of Consulting & Clinical Psychology | 5 |
| 35 | Hamdan | 2018 | Developing a Proposed Training Program Based on Discrete Trial Training (DTT) to Improve the Non-Verbal Communication Skills in Children with Autism Spectrum Disorder (ASD). | International Journal of Special Education | 4 |
| 36 | Estes | 2015 | Long-Term Outcomes of Early Intervention in 6-Year-Old Children With Autism Spectrum Disorder | Journal of the American Academy of Child and Adolescent Psychiatry | 5 |
| 37 | McDaniel | 2020 | Effects of pivotal response treatment on reciprocal vocal contingency in a randomized controlled trial of children with autism spectrum disorder | Autism | 4 |
| 38 | Kasari | 2006 | Joint attention and symbolic play in young children with autism: a randomized controlled intervention study | Journal of Child Psychology and Psychiatry | 4 |
| 39 | Gulsrud | 2016 | Isolating active ingredients in a parent-mediated social communication intervention for toddlers with autism spectrum disorder | Journal of Child Psychology and Psychiatry | 4 |
| 40 | Dawson | 2012 | Early Behavioral Intervention Is Associated With Normalized Brain Activity in Young Children With Autism | Journal of the American Academy of Child and Adolescent Psychiatry | 4 |
| 41 | Denne | 2018 | Common approaches to intervention for the support and education of children with autism in the UK: an internet-based parent survey | International Journal of Developmental Disabilities | 5 |
| 42 | Lee | 2020 | Perception of Parents and Special Education Teachers Analyst on Intervention of Applied Behavior Analysis on Children with ASD | Journal of Emotional & Behavioral Disorder | 5 |
| 43 | Kingsdorf | 2020 | A survey of the use of applied behaviour analysis for children with autism in the Czech Republic | European Journal of Special Needs Education | 5 |
| 44 | Lee | 2017 | A Survey on the Status of Hospital-Based Early Intensive Intervention for Autism Spectrum Disorder in South Korea | Journal of the Korean Academy of Child and Adolescent Psychiatry | 5 |

**eTable 3. Risk of Bias – Assignment to intervention (the ‘intention-to-treat’ effect)**

|  | **Randomization process** | **Deviations from intended interventions** | **Mising outcome data** | **Measurement of the outcome** | **Selection of the reported result** | **Overall Bias** |
| --- | --- | --- | --- | --- | --- | --- |
| Assignment to intervention (the 'intention-to-treat' effect) | | | |  |  |  |
| Total number of study = 16 | |  |  |  |  |  |
| Low risk | 56.3 | 0 | 81.3 | 93.8 | 93.8 | 0 |
| Some concerns | 31.3 | 12.5 | 12.5 | 0 | 6.3 | 0 |
| High risk | 12.5 | 87.5 | 6.3 | 6.3 | 0 | 100 |

**eTable 4. Conflict of Interest Evaluation**

| No. | Author | Year | Funding Source | COI Declaration Content | Remarks |
| --- | --- | --- | --- | --- | --- |
| 1 | Chang | 2016 | Autism Speaks Grant #7495 (PI: Kasari) | None | Declared no competing interests in the paper. |
| 2 | Dawson | 2010 | National  Institute of Mental Health grant  U54MH066399 (to Dr Dawson) | Sally Rogers and Geraldine Dawson  are authors of Early Start Denver Model for Young Children with  Autism from which they receive royalties. | Authors declared receiving royalties from the intervention model. |
| 3 | deKorte | 2021 | Fonds NutsOhra (project 1405-307); Karakter; EU-AIMS; AIMS-2-TRIALS (Grant No. 115300 & 777394); European Union Horizon2020 programme CANDY (Grant No. 847818) | Jan K. Buitelaar has been in the past three years a consultant to/member of the advisory board of/and/or speaker for Takeda/Shire, Roche, Medice, Janssen Cilag, Angelini, and Servier. He is not an employee of any of these companies, and not a stock shareholder of any of these companies. He has no other financial or material support, including expert testimony, patents, and royalties. All other authors declare that they have no conflict of interest. | One author (Jan K. Buitelaar) declared multiple affiliations and consulting roles with pharmaceutical companies; other authors declared no conflict of interest. |
| 4 | Dixon | 2021 | Not reported in the publication | First author receives small royalties from sales of the PEAK curriculum. Remaining authors declare they have no conflict of interest. | The first author has a financial interest in the PEAK curriculum used in the study. |
| 5 | Gengoux | 2019 | National Institute on Deafness and Other Communication Disorders (DC01368902; Dr Hardan);  a National Institute of Mental Health (K01 Mentored Research Scientist Development Award MH102428; Dr  Abrams); National Center for Research Resources and the National Center for Advancing Translational Sciences; National Institutes of Health through  grant UL1 TR001085; National Institutes of Health (NIH). | POTENTIAL CONFLICT OF INTEREST  Dr Frazier is employed by Autism Speaks; the other authors have indicated they have no potential conflicts of interest to disclose.  FINANCIAL DISCLOSURE  The authors have indicated they have no financial relationships relevant to this article to disclose | One author (Dr. Frazier) is employed by Autism Speaks. Other authors declared no financial conflicts of interest related to the article. |
| 6 | Goods | 2013 | Autism Research grant 2007272, Autism Speaks grant 5666, NIH/ NICHD 1 P50-HD-055784, and Department of Health and Human Services UA3MC11055. | Not reported in the publication | - |
| 7 | Hardan | 2015 | Autism Speaks Treatment Grant (5773; PI: Hardan); National Center for Research Resources and the National Center for Advancing Translational Sciences, NIH (grant UL1 RR025744) | The authors have declared that they have no competing or potential conflicts of interest. | Authors declared no competing or potential conflicts of interest. |
| 8 | Kasari | 2014 | Maternal and Child Health Research Program, Maternal and Child Health Bureau (Combating Autism Act Initiative), Health Resources and Services Administration, Department of Health and Human Services (grant UA3 MC 11055 AIR-B) | Dr Lord receives royalties from Western Psychological Services for diagnostic instruments (ADOS, ADI-R); royalties are given to a non-profit organization. The other authors have indicated they have no potential conflicts of interest to disclose. The authors have also indicated they have no financial relationships relevant to this article to disclose | One author (Dr. Lord) disclosed receiving royalties from diagnostic instruments, which are donated to a non-profit. Other authors stated no financial or potential conflicts of interest. |
| 9 | Kasari | 2015 | UA3 MC 11055 AIR-B from the Maternal and Child Health Research Program, Maternal and Child Health Bureau (Combating Autism Act Initiative), Health Resources and Services Administration, Department of Health and Human Services. | The authors have indicated they have no ﬁnancial relationships relevant to this article to disclose. | All authors declared no conflicts of interest |
| 10 | Lawton | 2012 | Not reported in the publication | Not reported in the publication | - |
| 11 | Mohammadzaheri | 2022 | Not reported in the publication | Dr. Lynn Koegel is a partner in the pri- vate frm Koegel Autism Consultants, LLC that provides PRT training. |  |
| 12 | Panganiban | 2022 | 3104 John & Marcia Goldman Foundation (PI: Kasari); UA3 MC11055 HRSA (PI: Kasari) | The author(s) declared no potential conflicts of interest with respect to the research, authorship, and/or publication of this article. | Authors declared no potential conflicts of interest. |
| 13 | Rogers | 2012 | Autism Speaks grants (A.E., S.R.); National Institute of Mental Health (NIMH)/National Institute of Child Health and Human Development (NICHD) grant MH R01 081757 (S.R.) | Drs. Rogers, Vismara, and Dawson have received royalties from the sale of books and other printed Early Start Denver Model (ESDM) materials. Drs. Estes, Lord, Winter, Fitzpatrick, and Guo report no biomedical ﬁnancial interests or potential conﬂicts of interest. | Key authors (Rogers, Vismara, Dawson) receive royalties from ESDM materials. Other authors declared no financial conflicts. |
| 14 | Rogers | 2019 | National Institute of Mental Health/Eunice Kennedy Shriver National Institute of Child Health and Human Development award number R01 081757 (Sally J. Rogers, principal investigator) as part of the Autism Centers of Excellence Treatment Network (clinicaltrials.gov, identiﬁer NCT 00698997), Autism Speaks grants 8089 (to Dr. Rogers) and 8376 (to Dr. Estes) | Drs. Rogers, Dawson, and Vismara have received royalties from Guilford Press for materials related to this work. Dr. Lord has received royalties from Western Psychological Services. All of Drs. Rogers' and Lord’s  Royalties related to this project were donated to charity. Drs. Estes, Munson, Rocha, Winter, Greenson, Colombi, Sugar, Hellemann, and Talbott and Ms. Whelan report no biomedical ﬁnancial interests or potential conﬂicts of interest. | Key authors (Rogers, Dawson, Vismara, Lord) received royalties from publishers (Guilford Press, Western Psychological Services); Rogers' and Lord's royalties were donated to charity. Other listed authors reported no financial interests or potential conflicts. |
| 15 | Sullivan | 2014 | Not reported in the publication | Not reported in the publication | - |
| 16 | Vernon | 2019 | Autism Speaks | All authors declare that they have no conflicts of interest | All authors declared no conflicts of interest |
| 17 | Andersen | 2017 | Not reported in the publication | No potential conflict of interest was reported by the authors | Authors declared no potential conflicts of interest |
| 18 | Bang | 2021 | Not reported in the publication | Not reported in the publication | - |
| 19 | Boyd | 2001 | Not reported in the publication | Not reported in the publication | - |
| 20 | Dillenburger | 2004 | Not reported in the publication | Not reported in the publication | - |
| 21 | Dillenburger | 2012 | Royal Irish Academy's Third Sector Research Program under an initiative called “Developing Charity Strategy Through Partnership.” | Not reported in the publication | - |
| 22 | Grindle | 2009 | Esme´e Fairburn Foundation | Not reported in the publication | - |
| 23 | McPhilamy | 2013 | Not reported in the publication | Not reported in the publication | Research was conducted in part fulfillment of the MScASD at Queen’s University Belfast for the first author. |
| 24 | Park | 2016 | 2016 of the Korea Nazarene University | Not reported in the publication | - |
| 25 | Tzanakaki | 2012 | Not reported in the publication | Not reported in the publication | - |

**eFigure 1. Results of quality assessment for RCTs.**


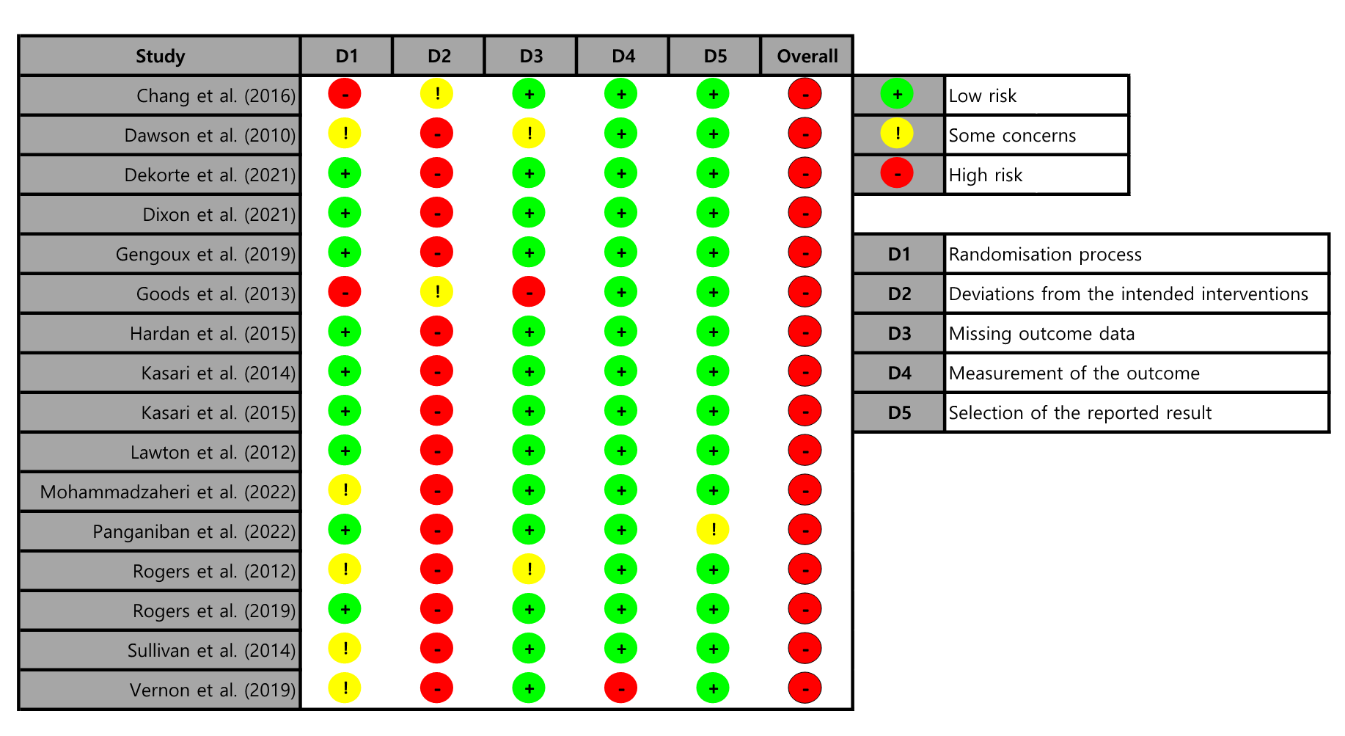


**eFigure 2. Results of quality assessment for qualitative studies.**


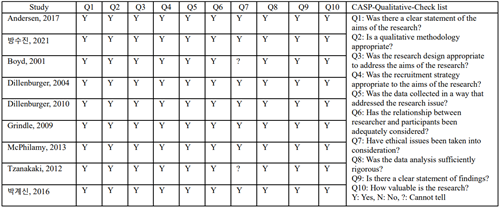


**eFigure 3. Baujet plot for language skills outcomes.**


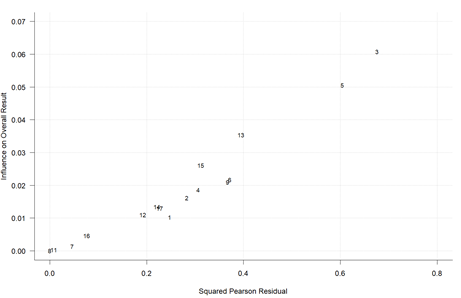


**eFigure 4. Funnel plots for each outcomes.**

**
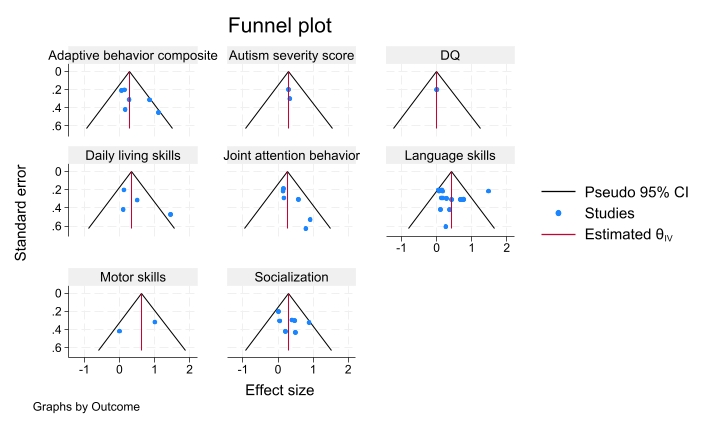
**
